# Emergency remote teaching in higher education: mapping the first global online semester

**DOI:** 10.1186/s41239-021-00282-x

**Published:** 2021-08-30

**Authors:** Melissa Bond, Svenja Bedenlier, Victoria I. Marín, Marion Händel

**Affiliations:** 1 EPPI-Centre, Institute of Education, University College London, London, UK; 2 Faculty of Humanities, Social Sciences, and Theology, Friedrich-Alexander-Universität Erlangen-Nürnberg, Erlangen, Germany; 3 Faculty of Education, Psychology and Social Work, Universitat de Lleida, Lleida, Spain

**Keywords:** Emergency remote teaching, Higher education, Systematic mapping review, Covid-19, Educational technology

## Abstract

Due to the Covid-19 pandemic that spread globally in 2020, higher education courses were subsequently offered in fully remote, online formats. A plethora of primary studies began investigating a range of topics exploring teaching and learning in higher education, particularly during the initial semester. In order to provide an overview and initial understanding of this emerging research field, a systematic mapping review was conducted that collates and describes the characteristics of 282 primary empirical studies. Findings reveal that research was carried out mostly descriptively and cross-sectionally, focusing predominantly on undergraduate students and their perceptions of teaching and learning during the pandemic. Studies originate from a broad range of countries, are overwhelmingly published open access, and largely focused on the fields of *Health & Welfare* and *Natural Sciences, Mathematics & Statistics*. Educational technology used for emergency remote teaching are most often *synchronous collaborative tools*, used in combination with *text-based tools*. The findings are discussed against pre-pandemic research on educational technology use in higher education teaching and learning, and perspectives for further research are provided.

**Supplementary Information:**

The online version contains supplementary material available at 10.1186/s41239-021-00282-x.

## Introduction

Globally, the first semester of 2020 marked a turning point within education; the Covid-19 pandemic lead to the unprecedented situation of having to switch to online instruction. Early on considered as *emergency remote teaching—ERT* (Hodges et al., 2020), it has turned – and continues to turn—teaching and learning upside down, with considerable impact on students in all levels of education (Bond, 2020; Marinoni et al., 2020).

During the pandemic, most higher education institutions deployed a strategy of ERT, which can be considered as a branch of distance education (Bozkurt et al., 2020; Hodges et al., 2020). The special feature of emergency remote education is that it is an unplanned practice, with no option than to use any kind of offline and/or online resources that may be at hand. Stemming from this situation, researchers from across the globe have started to investigate a broad variety of topics related to teaching and learning during the pandemic, including studies on, for example, how educators’ and students’ acceptance of digital formats changed in the context of Covid-19, and how this potentially affects higher education in the long-term (Vallaster & Sageder, 2020), experienced instructors’ views on online teaching and advice (Rapanta et al., 2020) or the relation between digital readiness and the social-emotional state of students (Händel, Stephan et al., 2020).

With the emergence of primary research and the accompanying focus on specific courses, institutions and populations, research is needed that provides orientation within this vast field, in an attempt to structure the presently growing body of knowledge. Initial research overviews have begun to emerge, ranging from the commented list of selected studies on Covid-19 and ERT (Bates, 2020), a Padlet to collate information and links to studies (Hochschuldidaktisches Zentrum Sachsen, n.d.), to the open access COVID-19 in Higher Education Literature Database (CHELD V1) (Butler-Henderson et al., 2020). The research presented in this article aligns with these first endeavours of providing an insight into the emerging field of research around ERT. It aims to provide a glimpse into the breadth and depth of higher education studies that have been conducted so far, focusing on teaching and learning in the first semester of 2020 (April–September), by systematically collating information on primary studies.

### Prior reviews of emergency remote research in higher education during the COVID-19 pandemic

A number of descriptive articles have been published that address the institutional processes that higher education institutions around the world implemented, in order to adapt to the pandemic, which provide useful lessons on failures and successes. For example, Bozkurt et al. (2020) analysed both the K-12 and higher educational landscape, covering 31 countries, and identified the main issues of concern in relation to the interruption of education, such as psychological pressure and anxiety, alternative assessment and evaluation methods, as well as surveillance and data privacy concerns. Crawford et al. (2020) also analysed 20 countries’ higher education intra-period digital pedagogy responses to COVID-19 and noted three typologies of response, ranging from no response through to social isolation strategies on campus, and rapid curriculum redevelopment for fully online offerings, including the extension of the semester break, campus closures and the move to online teaching. These higher education responses also involved diverse decisions regarding teaching and learning. By conducting a qualitative content analysis of 52 student surveys and 17 instructor surveys at higher education institutions in Germany, Arndt et al. (2020) derived 13 central topics across the institutions, for example, workload, communication and interaction, prior experience and the impact on courses, and the evaluation of the switch from in-person to online learning.

Apart from descriptive studies, a number of secondary reviews have been conducted. For instance, the previously mentioned open access COVID-19 in Higher Education Literature Database (CHELD V1; Butler-Henderson et al., 2020) represents a valuable resource to support research into literature on higher education during the pandemic. The thematic literature review by Bhuwandeep and Das (2020) identified three trends that emerged with emergency remote education during COVID-19: blended learning, access and availability to e-resources, and stakeholder theory in distance education. The bibliometric analysis by González-Zamar et al. (2021) identified the impact of returning to the classroom with the effects on the cognitive processes, motivations and academic performance of students as the main research trends on the effects of COVID-19 in university classrooms during the summer semester 2020. The discipline of medicine has emerged as the most prolific, with major clusters of collaboration in terms of co-authorship, observed by the type of studies in those cases and most common keywords identified in the included articles (González-Zamar et al., 2021).

Whilst we recognise that previous reviews have begun the process of collating higher education teaching and learning research undertaken during the pandemic (e.g. Butler-Henderson et al, 2020), this review uses a larger number of databases and includes articles written in three languages. Furthermore, this article represents only the first stage of this project, mapping the literature in the early stages of the pandemic. The next stage of this research will see the ongoing evolution of this work as an open access living review, in the hopes of providing the higher education community with a resource that provides multiple insights into the implications for research, policy and practice.

### Research questions

Against this background, the following research questions guide this mapping study:

1. Where, when and by whom has research on teaching and learning in higher education during the COVID-19 pandemic been published?
2. What are the characteristics of, methods used, and topics studied in teaching and learning research in higher education during the COVID-19 pandemic?
3. What technology has been used during emergency remote teaching in higher education?

## Methodology

In order to provide first insights into the rapidly emerging field of ERT in higher education, a systematic review was pre-registered (Händel, Bedenlier et al., 2020) and conducted using explicit and transparent methods (Gough et al., 2012; Zawacki-Richter et al., 2020), and guided by the PRISMA reporting guidelines (Page et al., 2020). This mapping article provides a first overview of the research that has been undertaken during the initial stages of the pandemic, located using pre-defined inclusion/exclusion criteria. This will also be a living systematic review (Elliott et al., 2014), which means that it will be regularly updated with new studies that are published during the pandemic, that meet the inclusion criteria. The living review will be publicly available (see Bond et al., 2021).

### Search strategy and study selection

The initial search was conducted on 24 July 2020, with subsequent searches conducted until the first week of December 2020. As the author team is trilingual, studies that were written in English or Spanish were targeted for potential inclusion, and studies that were found in German during the search were also considered. The platforms and databases searched for English language studies were Web of Science, Google Scholar, ERIC, PsycINFO, Scopus, ProQuest, EBSCOHost and Microsoft Academic Graph (see Chen, 2020), as well as the COVID-19 living systematic map (EPPI-Centre et al., 2021). For Spanish studies, Dialnet was searched, alongside Web of Science, Latindex, Redalyc and Google Scholar (Marín & Zawacki-Richter,  2019). These databases were chosen, as they are considered well-suited to evidence synthesis, with the Web of Science, Scopus and EBSCOHost, for example, being found particularly useful in a recent review (Gusenbauer & Haddaway, 2019). A number of studies were also identified during the life of the review through, for example, special issues being published, studies being published on Twitter, or through the COVID-19 research community on ResearchGate.^1^ Additionally, empirical studies included in the CHELD V1 database as of September 9, 2020 were included (Butler-Henderson et al., 2020) if they met the inclusion criteria and had not been duplicates of our own search. Searching pre-print servers and grey literature has been recommended when searching for research undertaken during the pandemic (e.g., Tricco et al., 2020), due to peer review duration and the “rapidly changing nature of the research landscape” (Bond, 2020, p. 195).

### Search string

Two search strings were developed, one for each language (see Tables 1 and 2), focusing on formal teaching and learning settings in higher education during the pandemic, and using * for truncations. Given the large amount of medical studies published during the COVID-19 pandemic (see EPPI-Centre et al., 2021), medical terms were added as ‘NOT’ terms, such as ‘pathology’, ‘telemedicine’ and ‘inflammation’, in order to further refine the search results, especially in the English search.

**Table 1:** Search string, English language studies

“Emergency remote teaching” OR “student-centred remote teaching” OR “emergency remote education” OR “student-centered remote teaching” OR “COVID-19” OR “COVID19” OR pandemic OR “Corona virus” OR “online pivot”
AND
Universit* OR “higher education” OR postgrad* OR undergrad* OR “tertiary education” OR college
NOT
Pharmaceutical OR pharmacy OR clinic* OR pathology OR telemedicine OR telehealth OR inflammation OR patient* OR neurolog* OR surgery

**Table 2:** Search string, Spanish language studies

“Enseñanza remota de emergencia” OR “educación remota de emergencia” OR “docencia no presencial de emergencia” OR “docencia virtual” OR “COVID-19” OR “COVID19” OR “coronavirus” OR “pandemia”
AND
“Educación superior” OR “estudio* universitario*” OR “programa* universitario*” OR universitari*

### Inclusion/exclusion criteria

**Fig. 1:**
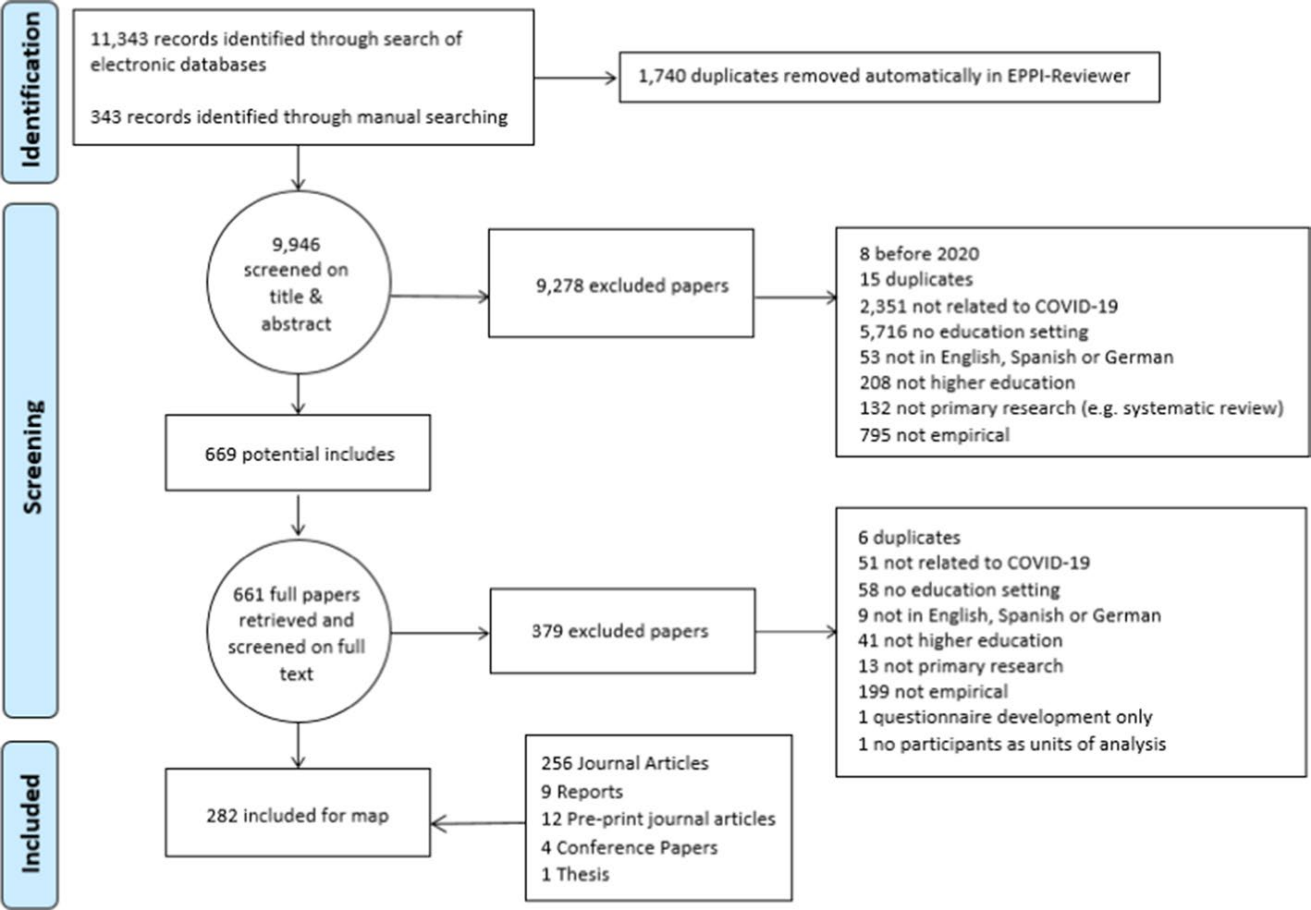
Systematic review PRISMA diagram

The combined strategy of searching electronic databases, websites, social media and organisations, yielded 11,686 items (see Fig. 1), which were imported into EPPI-Reviewer evidence synthesis software (Thomas et al., 2020). Following the automatic removal of 1,740 duplicates, 9946 items were screened on title and abstract by the four authors, applying the inclusion/exclusion criteria (see Table 3). Studies were included if they were empirical, written in English, German or Spanish, and explored teaching and learning in higher education during the COVID-19 pandemic (after January 2020).

**Table 3:** Inclusion and exclusion criteria

Inclusion criteria	Exclusion criteria
Higher education	K-12, further education
Teaching and learning setting (students, educators, administrators)	No teaching and learning setting
English, German or Spanish language	Not in English, German or Spanish
Empirical study	Not empirical or primary research
Studies undertaken during the COVID-19 pandemic	Studies undertaken before the outbreak of COVID-19
Studies published after January 2020	Published before 2020
Students, educators or administrators as unit of analysis	Unit of analysis not students, educators or administrators

In order to ensure inter-rater reliability between the four reviewers and authors of this study, the review team spent a considerable amount of time intensively discussing the codes and their meaning. Five rounds of comparison coding were conducted with 492 studies (100, 100, 92, 100 and 100), resulting in substantial agreement (Cohen’s k = 0.80) (McHugh, 2012). Following this, 9946 items were screened on title and abstract, resulting in 669 potential includes. During this process, the reviewers adjusted the inclusion criteria to specifically ensure that only studies with students, educators or administrators as the units of analysis would be included.

Given the breadth of studies included for consideration, further rounds of screening to calibrate mutual agreement on the inclusion criteria were undertaken at the screen on full text stage. After retrieving 661 items to screen on full text, seven rounds of calibration and reconciliation were conducted (20, 20, 20, 20, 50, 100, and 140 items), resulting in strong agreement (Cohen’s k = 0.83), and 282 studies being included for the initial map. Please note, however, that this is a living review, with further studies to be added in the future. Researchers are encouraged to contact the authors, with suggestions of research for possible inclusion. It should also be noted that over 200 studies have already been identified for potential inclusion, since the writing of this article began, and those that match the inclusion criteria will be made available within the living review (Bond et al., 2021).

### Data extraction

Data extraction codes for this initial mapping stage included publication and study characteristics (e.g. publication name, participant focus, study level of students), methodology (e.g. study design, date of data collection), as well as research focus and technology used (research scope and the type of technology used, based on Bower’s, 2016 typology, see Additional file 2: Appendix S2). This coding system is a slightly modified version of the one used by Bond (2020) in order to extract data within EPPI-Reviewer (Thomas et al., 2020). An initial five studies were coded by all four authors, in order to ensure agreement on the coding scheme. A full list of the coding scheme is available online from ResearchGate.^2^

### Data synthesis

In order to provide an insight into the heterogeneous articles included within this review, a mapping approach was undertaken (Petersen et al., 2015, including a tabulation of the included studies’ characteristics (see Additional file 1: Appendix S1), in order to provide an overview of the research area, and to provide guidance of what has been researched and where gaps exist. Further tables are also provided throughout the text, or included as appendices, accompanied by a narrative description that summarises the results and frames the recommendations provided. However, further synthesis will be undertaken in the future, using the bioecological model of student engagement by Bond and Bedenlier (2019), in order to delve deeper into how the ERT approaches used during the pandemic affected teaching and learning.

### Interactive evidence gap map development

So as to provide an open and publicly accessible resource of research undertaken during the pandemic, interactive evidence gap maps were produced for each research question, using the EPPI-Mapper application (Digital Solution Foundry & EPPI-Centre, 2020). Following data extraction in EPPI-Reviewer (Thomas et al., 2020), a JSON report of all included studies was imported into EPPI-Mapper, where display and filter options were chosen.^3^ The HTML files of each map were saved, and are available to access and download (Bond et al., 2021). The interactive evidence gap maps provide users with the opportunity to explore cross tabulations of data within the review, beyond that which is provided within this article. Instructions are also provided within the ‘About’ section of each map, as to how other researchers can contact the author team, to suggest possible studies for inclusion in the living review (see Bond et al., 2021).

### Computer-assisted content analysis

In order to help answer research question two and provide further insight into the topics explored within publications during the pandemic, the content analysis software Leximancer^4^ was used. The popularity of computer-assisted content analysis methods has been growing in the past decade, particularly within the field of educational technology (e.g., Bond et al., 2019; Bozkurt, 2020; Marín et al., 2017, 2018; Zawacki-Richter & Latchem, 2018) It has been found to be particularly useful in refining qualitative findings, assisting the identification and understanding of connected themes (Lemon & Hayes, 2020), and is considered both an effective and efficient method of analysing data (Fisk et al., 2012; Krippendorff, 2013).

The title and abstracts of all included English language studies (*n* = 262) were converted into an Excel.csv file and imported into Leximancer. The decision was made to include English language only studies, due to how the software works. Stop words were removed (‘conducted’, ‘due’, ‘during’, ‘participants’, ‘reported’, ‘results’, ‘use’, ‘used’, ‘using’), and plural phrases were merged (e.g. ‘student’ and ‘students’). The software then automatically identified significant themes and concepts within two sentence blocks, and a concept map was produced (theme size of 50%), with the frequency and connectedness of identified concepts highlighted (Smith & Humphreys, 2006). Key themes were automatically produced in the concept map (e.g. students), due to the frequency and connectedness of the words within the data. The map was then analysed by the authors, involving cross-checking the map with the included studies, to ensure deeper understanding of the themes identified (Harwood et al., 2015).

### Methodological limitations

This mapping article represents a first attempt to systematically locate, categorise and analyse research that has been undertaken in higher education teaching and learning during the COVID-19 pandemic. Four large international databases were searched, as well as further repositories containing grey literature, and although articles written in both English and Spanish were explicitly searched for, and appropriate German language studies included, the search needs to be continuously updated, for example also including databases such as the Germany-based FIS Bildung,^5^ which now (as of April 2021) lists many more German language resources than were initially available. Furthermore, numerous journals have announced special issues on the impact of COVID-19 on teaching and learning, due to be published in 2021 (e.g. *Journal of Research on Technology in Education*, *Journal of Engineering Education*), as well as institutional evaluations and surveys (Arndt et al., 2020). These now available sources of research will need to be included in future iterations of the search strategy. Studies were included in case they met the pre-defined criteria for inclusion. This resulted, however, in having studies in the corpus that were published in journals considered as potentially predatory according to the journal inventory in Beall’s List.^6^ This was the case for six journals. Still, whilst this does not automatically indicate faulty research, considering the specific outlet of a study will be taken into consideration more closely in future iterations.

The review was conducted by four reviewers and, whilst attempts were made to reduce bias and inconsistency (see Section “Inclusion/exclusion criteria”), the possibility of the human flaw of having overlooked or misinterpreted information cannot be fully discounted. Furthermore, whilst it is important to conduct a quality appraisal of studies included within a systematic review (Harden & Gough, 2012), it was decided to map the available studies at this stage, prior to conducting further synthesis and quality assessment in future iterations of the review. We did, however, code many methodological aspects, and have provided recommendations for future research going forward (see Section “Discussion”).

## Findings

### Publication characteristics

The 256 published journal articles in this review were sourced from 155 unique journals (see Additional file 3: Appendix S3) from a range of disciplines (e.g., Health Sciences, Social Sciences). The *Journal of Chemical Education* published 36 articles, this being by far the highest number of articles sourced from one outlet. This is likely due to that journal opening a special issue call for papers in April 2020 that was published online in August.^7^ The remaining studies were pre-prints (*n* = 12), reports (*n* = 9), conference papers (*n* = 4) and one thesis. The vast majority of studies (88.3%, *n* = 249) are available open access, corresponding to the share of open access publication of K-12 research undertaken during the pandemic (Bond, 2020).

#### Where, when and by whom were studies published?

**Fig. 2:**
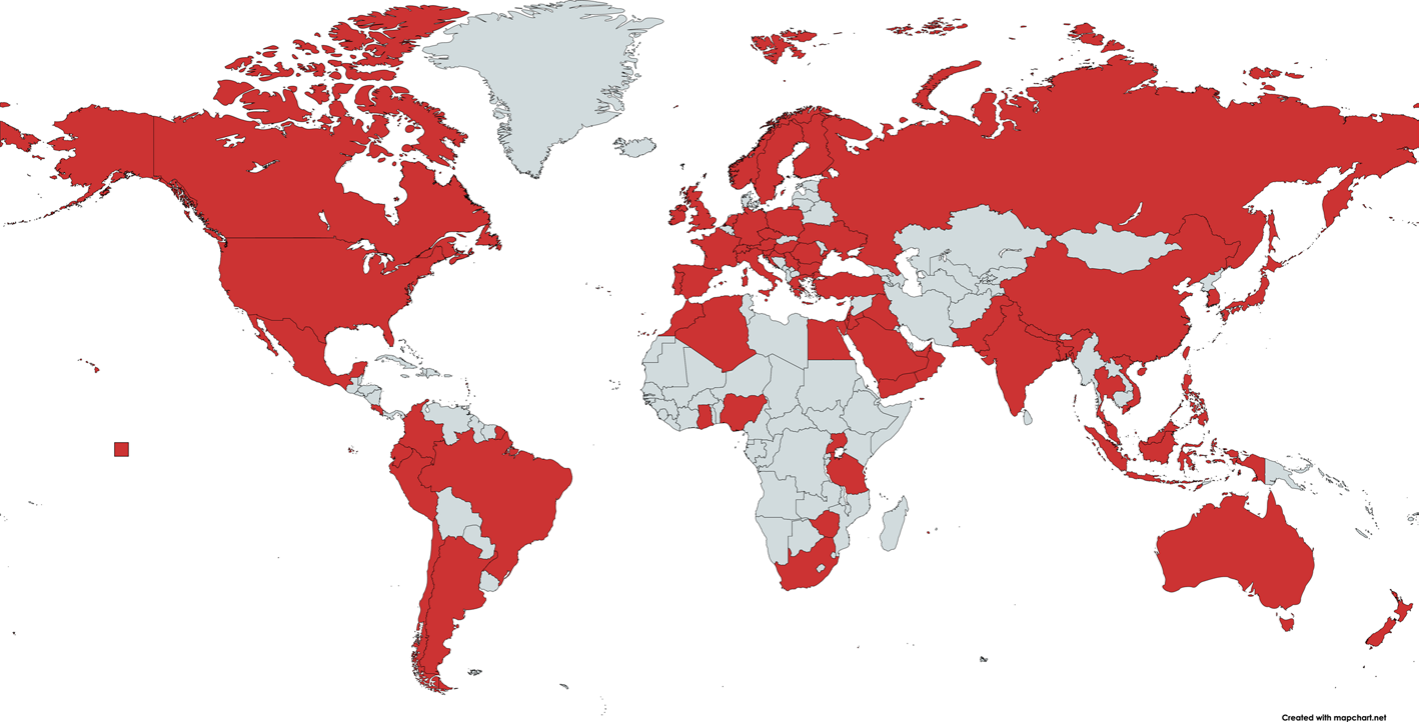
Geographical location of authors, created using https://mapchart.net/world.html

The studies in this review were published by 1,019 authors, mostly in teams of two or three (see Table 4), and hailing from 73 different countries (see Fig. 2), which covers a broader range of affiliation countries compared to the K-12 review (Bond, 2020). 10.3% (*n* = 29) included more than 6 authors, which was predominantly the case for studies where the discipline of the first author was either *Health & Welfare* or *Natural Sciences, Mathematics & Statistics*.

**Table 4:** Scope of article authorship (*n* = 282)

Number of authors	*N* studies	*N* studies [%]
1 author	50	17.7
2 authors	59	20.9
3 authors	56	19.9
4 authors	44	15.6
5 authors	30	10.6
6 authors	14	5.0
More than 6 authors	29	10.3

In terms of country affiliation, the United States was the most prevalent country (23.4%, *n* = 66), followed somewhat surprisingly by Saudi Arabia (7.4%, *n* = 21), Indonesia (6.4%, *n* = 18), India (6.0%, *n* = 17), Spain (5.7%, *n* = 16) and the United Kingdom (4.6%, *n* = 13). Most of the authors’ affiliation countries are located in Europe (27.7%), Asia (27.7%) and North America (25.5%), followed by the Middle East (15.6%), with little representation from South and Central America (5.7%), Africa (5.3%), and Oceania (2.5%); a finding that echoes prior educational technology in higher education research (e.g., Bond et al., 2019).

Most of the collaboration between authors were of a domestic only nature (68.8%, *n* = 194), which can also be understood by the type of articles that have been published and are included in this review, as most of them focus on the specific situation for teaching and learning in higher education within their institution and/or country (see e.g., Bozkurt et al., 2020). 17.4% were written by only one author, 10.3% were collaborations between international and domestic authors (two or more authors from the same country), and only 3.5% were published by international collaborations of authors from two or more completely different countries.

**Fig. 3:**
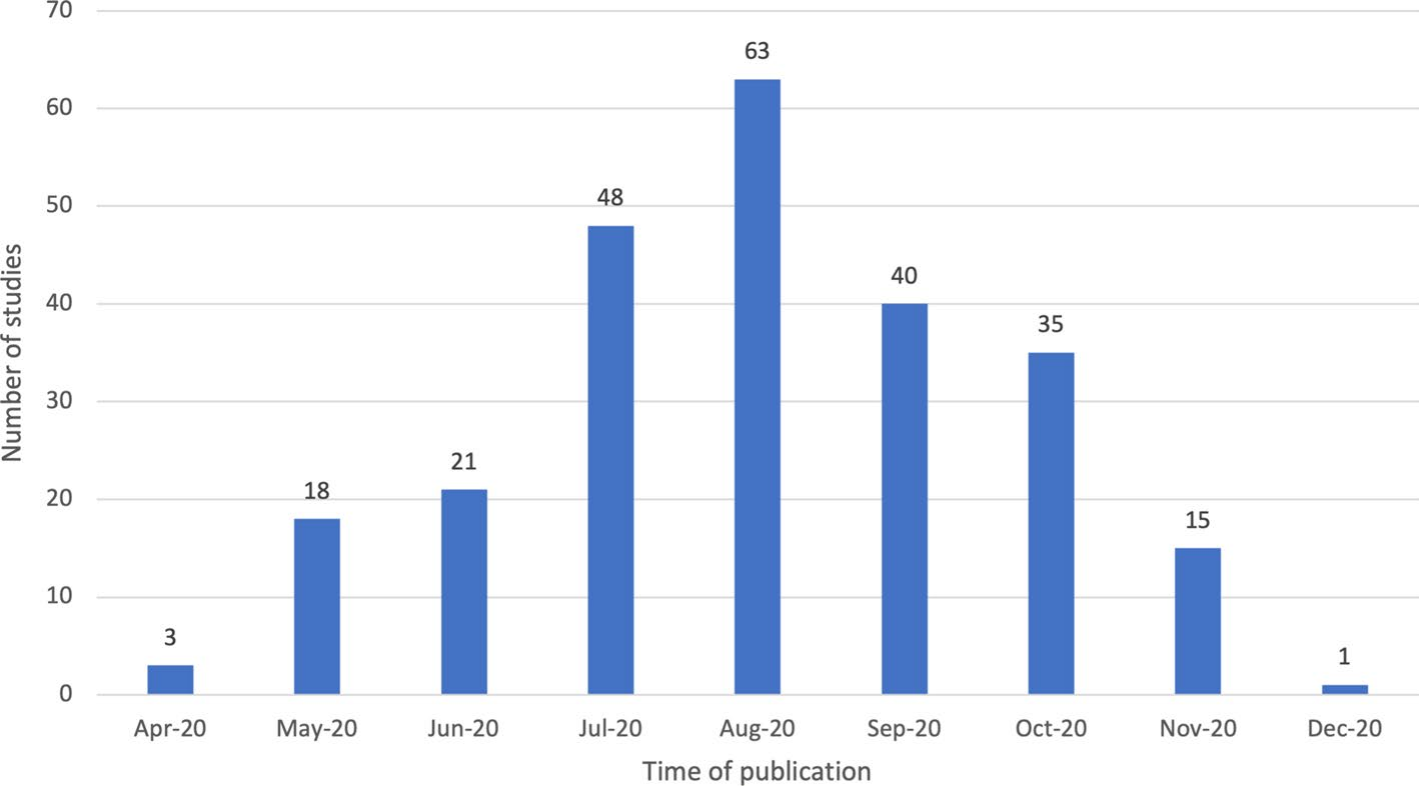
Timeline of study publication (*n* = 282)

Similar to the K-12 review (Bond, 2020), there was a first wave of publications that peaked in August 2020 (see Fig. 3). After September 2020, the number of monthly publications decreased. Unfortunately, however, it was not possible to identify the exact month of publication in 2020 for 38 studies. Looking at the discipline of the first author, all of them were represented, but the most frequent was *Health & Welfare* (22.3%, *n* = 63), followed by *Education* (18.1%, *n* = 51) and *Natural Sciences, Mathematics & Statistics* (16%, *n* = 45). For 36 studies (12.8%) it was not possible to identify the discipline of the first author.

#### Study characteristics

##### Geographical characteristics

Comparing both continental and country-level origin of authors and study participants, both correspond clearly and unsurprisingly. Participants mostly hailed from Asia (*n* = 78, 27.7%), North America (*n* = 64, 22.7%) and Europe (*n* = 77, 27.3%), followed by 14.2% studies from the Middle East (*n* = 40), 6.4% from South and Central America (*n* = 18), 6.0% from Africa (*n* = 17), and, finally, 2.1% from Oceania (*n* = 6). 1.1% of studies (*n* = 3) can be considered as global studies, that is, students or educators from many countries across the globe participated (e.g., Aristovnik et al., 2020).

A total of 79 individual countries are represented in the corpus, exceeding that of author affiliation by six countries. In 20.6% of studies, participants came from the USA (*n* = 58), participants from India are present in 7.4% studies (*n* = 21), followed by 6.4% studies with Indonesian participants (*n* = 18), 6.0% with Spanish (*n* = 17) and 5.7% with Saudi Arabian participants (*n* = 16). The UK follows with 4.6% studies (*n* = 13) and China with 3.2% studies (*n* = 9). However, it needs to be noted that 25 studies published in the *Journal of Chemical Education* all sourced their participants from the USA, causing this number to be dominant in comparison with other countries.

##### Sample focus

Most of the studies (68.1%, *n* = 192) focused solely on the experiences and perspectives of higher education students (see Additional file 4: Appendix S4), especially undergraduates (46.1%, *n* = 130). Only 36 studies focused just on teachers/instructors (12.8%), although there were studies that triangulated data between student and educators (12.1%, *n* = 34; e.g. Abdulrahim & Mabrouk, 2020), teachers and department managers (*n* = 4; e.g. Ahmed, 2020), or teachers and support staff (*n* = 2; e.g. Littlejohn, 2020). Other combinations were rare (e.g., IT experts and developers, students, teachers and policy makers, *n* = 1) or non-existent (librarians with no other groups, *n* = 2). However, numerous studies included more than one group of participants, with the combination of students and instructors/teachers being the most frequent.

The studies were also categorised according to their sample size. While 25.2% of the studies can be considered large, with more than 400 participants, including a number of institutional (e.g., Alturise, 2020) and international (e.g., Aristovnik et al., 2020; Elumalai et al., 2020) surveys, but particularly comprised of national surveys (e.g., Wang et al., 2020), 16.3% of studies in the review had sample sizes of up to 25 participants (see Additional file 5: Appendix S5). This is in stark comparison to studies of K-12 teaching and learning during the pandemic, with 34% of studies focusing on 25 or fewer respondents (Bond, 2020).

##### Discipline and education setting

In order to allocate participants’ study discipline to fields of study, the ISCED classification (UNESCO, 2015) was used. In instances where participating students and instructors stemmed from a number of fields of study, all disciplines were marked (see Table 5), and it should be noted that each study could include more than one discipline (see Additional file 6: Appendix S6). The most researched disciplines were *Health & Welfare* (27.3%), followed by *Natural Sciences, Mathematics & Statistics* (24.1%) and *Education* (16%). For 23.4% of the studies, the discipline of participants was unclear.

**Table 5:** Researched disciplines in the corpus (*n* = 282)

Discipline of Participants	*N* studies	*N* studies [%]
Health & Welfare	77	27.3
Natural Science, Maths & Statistics	68	24.1
Unclear	66	23.4
Education	45	16.0
Arts & Humanities	40	14.2
Engineering, Manufacturing & Construction	30	10.6
Business, Administration & Law	26	9.2
Social Sciences, Journalism & Information	26	9.2
ICT	16	5.7
Other	2	0.7
Agriculture, Forestry, Fisheries and Veterinary	2	0.7

In 40.8% of studies (*n* = 115), research was conducted with participants who were not sourced from a specific course, discipline or department, but generally investigated perceptions of teaching and learning in summer 2020, followed by studies within a specific department (21.6%, *n* = 61). Course-specific research accounts for 26.6% (*n* = 75) of the studies, and 6.4% of the studies were conducted within a discipline or a specific study program (5%).

#### Methodological characteristics

To provide an overview of the methodological characteristics of the studies, information was extracted about their approach, study design, data collection methods, when the studies had been performed (date of data collection), and the type of data analysis performed. It should first be noted, however, that 18.8% of studies did not appear to formulate any aims, research questions, or hypotheses. Furthermore, mirroring prior educational technology research (e.g., Hew et al., 2019), only 10.6% (*n* = 30) of studies in this sample used a theoretical framework, with the most used the Technology Acceptance Model (Davis, 1989), such as the Indonesian study by Sukendro et al. (2020), which used an expanded model to explore Indonesian students’ use of educational technology during the pandemic. Hence it could be said that, not only was the research focused on emergency remote teaching and learning, but also the design and conduct of the research itself could be considered as “emergency remote research” (Bond, 2020, p. 202).

##### Approach and study design

The majority of studies were coded as quantitative research (*n* = 151, 53.6%), 16.3% of studies (*n* = 46) used a qualitative approach, and 30.1% (*n* = 85) were coded as mixed methods studies. The vast majority were cross-sectional studies (92.2%, *n* = 260) and thereby provide an overview on the situation under consideration. In contrast, the studies with a longitudinal study design (5.3%, *n* = 15), have the potential to display developments and to investigate reciprocal effects (e.g. Knudson, 2020; Wang & East, 2020). For example, some studies had data available from previous study terms and could track students’ development (e.g., Klegeris, 2020). In addition, 2.5% of studies used (quasi-)experimental designs to detect group differences regarding specific interventions (e.g. Gonzalez et al., 2020; Sáiz-Manzanares et al., 2020).

Sample sizes seemed to be equally distributed among the different study designs; i.e., there were different sample sizes across cross-sectional, longitudinal or (quasi-)experimental research, indicating that large (institutional) surveys with more power, as well as small cross-sectional studies, were conducted.

##### Data collection and analysis

**Fig. 4:**
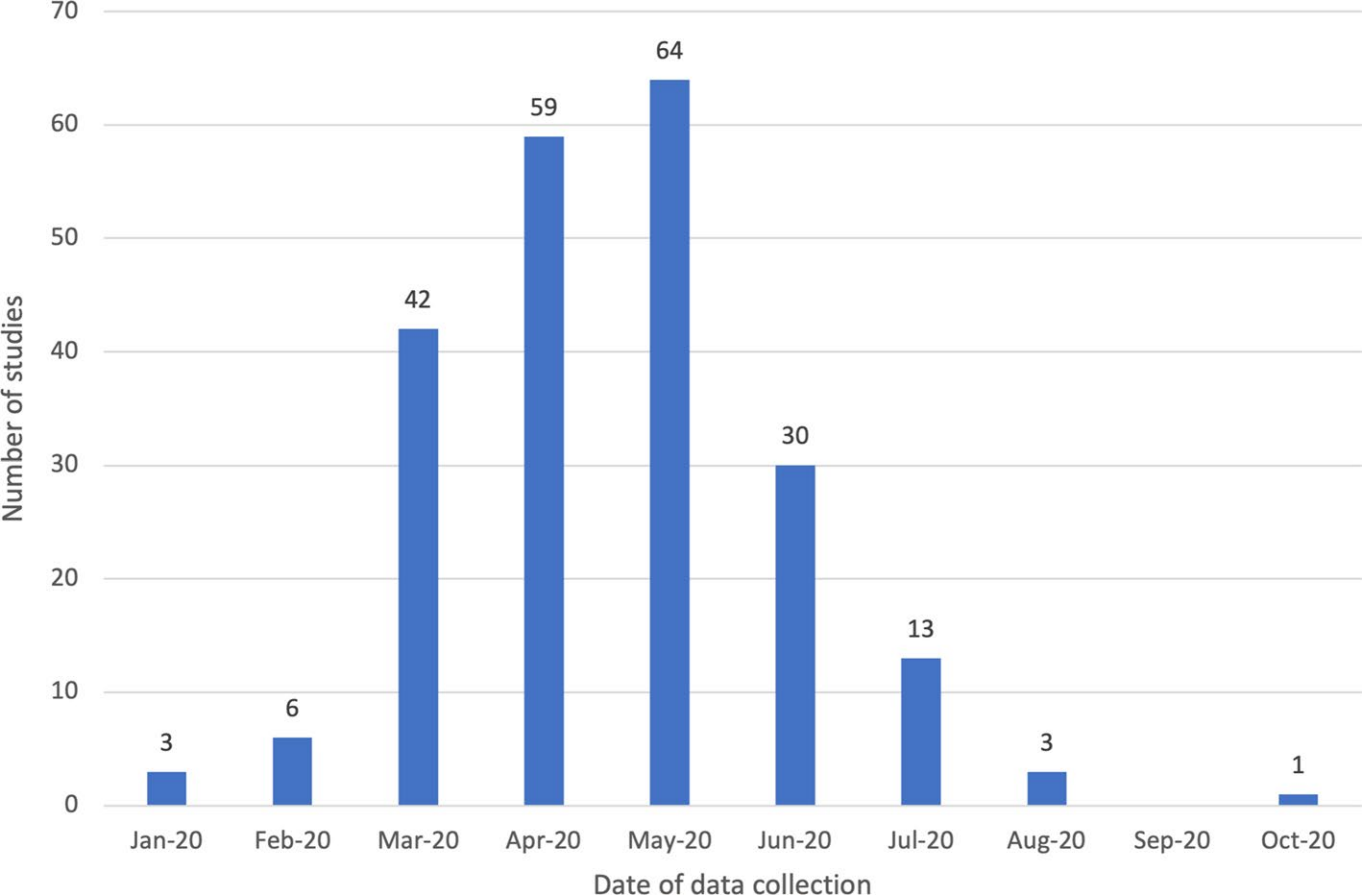
Data collection timeline

The current review encompasses studies published between January 2020 and October 2020 (see Fig. 4). Most of those studies that reported when data were collected, had done so between March 2020 and June 2020, that is at the very beginning of the shift to online teaching and 2020 learning. However, more than half of the studies included in the review (55.7%, *n* = 157) did not include information as to when their data were collected. Given the variable spread of COVID-19 throughout the world, it is important to frame study results within this kind of contextual information (Bond, 2020).

Due to the situation of ERT, many students could not be on-campus, and could only participate in studies via online measures. Accordingly, it is not surprising that the majority of studies (83%, *n* = 234) used online surveys for their research (see Additional file 7: Appendix S7), followed by interviews (14.5%) and students grades (7.4%). The most often combined methods of data collection were surveys and interviews (8.5%, *n* = 24), such as the study of Indian educators and postgraduate students by Mishra et al. (2020), followed by surveys and student grades (7.4%, *n* = 21), such as the study of Canadian undergraduate Chemistry students by Rodríguez Núñez and Leeuwner (2020).

Four in every five studies (*n* = 227) reported using descriptive statistics to analyse their data (see Additional file 8: Appendix S8), while a smaller proportion of studies reported correlational or inferential statistics. This indicates that many studies can be considered as descriptive studies on the status quo of emergency remote teaching and learning. A small proportion of studies aimed to develop new measures and were explicitly concerned with psychometric analyses (e.g., Dwidienawati et al., 2020). 30.5% of studies employed qualitative data analyses, with a broad range of analysis approaches (e.g., content analysis, auto-narrative analysis), however 8.2% did not explicitly mention in their report, how they undertook their analysis.

###### Terminology used about research on teaching and learning during the pandemic

The terminology used to describe ERT throughout the studies varied greatly, with 71 individual terms used (see Additional file 9: Appendix S9). Across the study corpus, ‘online learning’ was the most frequently used term (20.6%, see Table 6), with ‘emergency remote teaching’ being used relatively less often (5.3%), which was surprising given its popularity amongst the educational technology community (e.g., Bozkurt et al., 2020; Hodges et al., 2020). However, the terms coded refer to terms and concepts that are well-established in the literature, with some studies opting to use multiple terms.

**Table 6:** Top 10 used terms for online learning during the pandemic

Terminology used	*N* Studies	*N* Studies [%]
Online learning	58	20.6
e-Learning	52	18.4
Distance learning	50	17.7
Online teaching	33	11.7
Online education	18	6.4
not specified	17	6.0
Internet Web-Based Learning	16	5.7
Emergency remote teaching	15	5.3
Remote learning	15	5.3
Computer-Based Learning	8	2.8
Distance education	8	2.8

Some studies also coined terms such as emergency remote online learning (e.g. Jeffery & Bauer, 2020), digital higher education (Littlejohn, 2020), home learning (Schmölz, Geppert, & Barberi, 2020) or multimedia-based learning (Scruggs et al., 2020). Others referred directly to specific forms of online learning and teaching, mostly when using terms related to assessment; examples include e-assessment (Sharadgah & Sa’di, 2020) or remote E-exams (Elsalem et al., 2020). As the terminology was derived from either the wording in the title, abstract, keywords or research questions, it needs to be noted that, for example, the *Journal of Chemical Education* used predefined keywords such as Internet Web-Based Learning and Distance Learning, which is presumably one reason why these terms were found with such frequency.

### Study focus

The studies that were published in the first semester of the pandemic were predominantly focused on student perceptions of emergency remote education and online learning (see Table 7), followed by the impact of the shift to ERT and teacher perceptions of online learning during the pandemic. Surprisingly little research focused on student (4.6%) and educator well-being (1.1%), or on teacher professional development (1.1%). Only two studies focused specifically on students with special education needs and disabilities (SEND), with Alsadoon and Turkestani (2020) reporting on the experiences of teachers of hearing-impaired students, and Bartz (2020) exploring the experiences of students with disabilities and mental disorders at German universities.

**Table 7:** Top five topic focus of studies (*n* = 282)

Area of focus	*N* studies	*N* studies [%]
Student perceptions of online learning	171	60.6
Impact of shift to online learning	84	29.8
Teacher perceptions of online learning	54	19.1
Students’ technical equipment	38	13.5
Course redesign	31	11.0

**Fig. 5:**
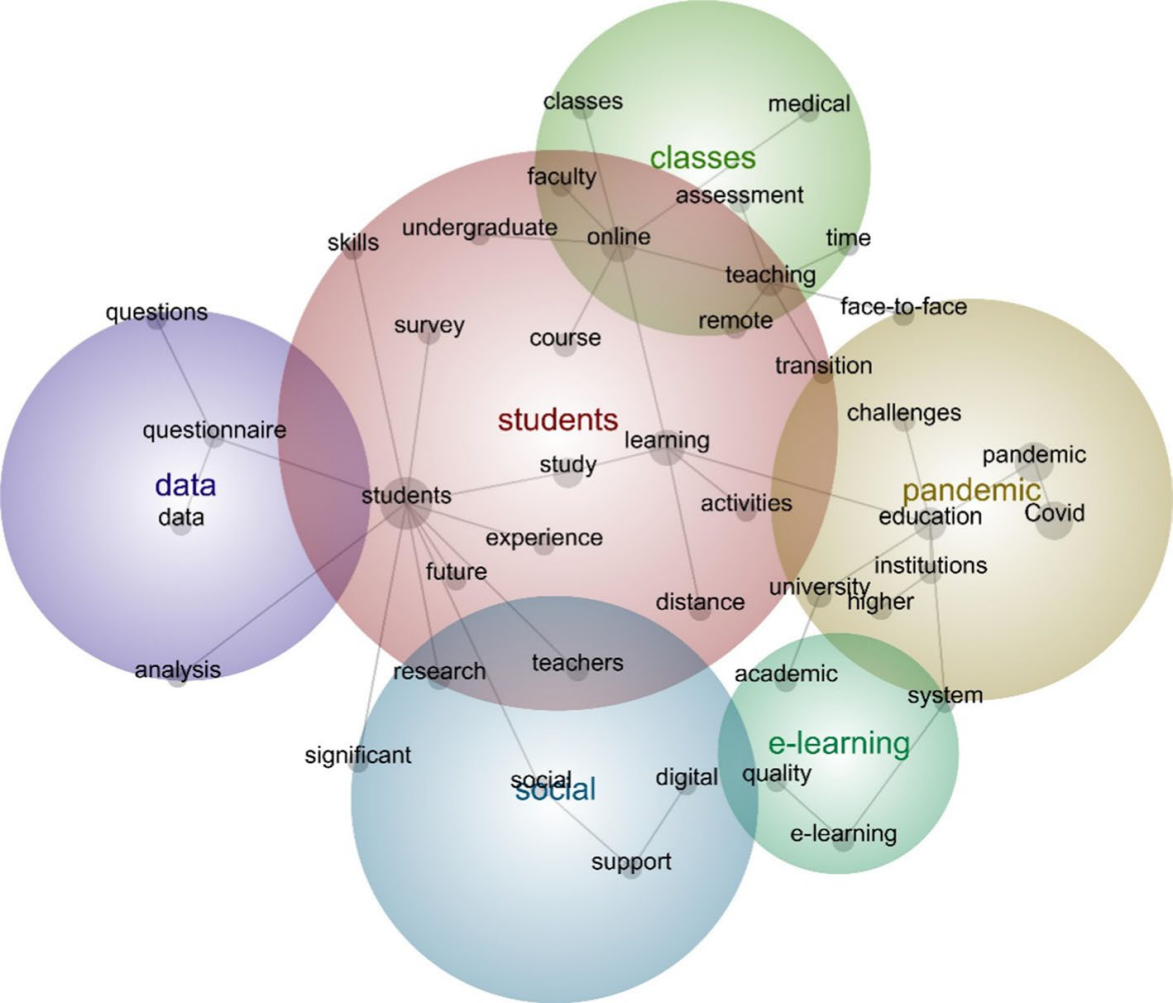
Concept map of study titles and abstracts (*n* = 262)

In order to gain further insight into the breadth of topics explored within the review corpus, a concept map was produced using content analysis software Leximancer (see Fig. 5). The thematic summary reveals that *students* has the most direct mentions with 1343 (100% relative count), followed by *pandemic* (52% connectivity), *e-learning* (7%), *classes* (7%), *social* (7%) and *data* (6%). The map confirms the findings of the topic analysis, indicating that higher education research has heavily focused on the experiences of students during the pandemic (see *experience-students-study-learning-activities*), and particularly that of undergraduate and medical students (see *undergraduate-online-learning-activities* and *students-learning-online-medical*). The map further reveals that research has sought to explore the quality of online teaching and learning (*quality-e-learning-system-institutions-education-challenges*), as well as how students could be supported through digital technology during the pandemic (see *students-social-support-digital*). Interestingly, the map indicates a focus on assessment (see *students-learning-online-teaching-assessment*), although the topic analysis only found 10% of studies with assessment as a research focus; a potential area for further exploration.

#### Technology use for emergency remote teaching

Based on Bower’s (2016) typology, we identified and coded 12 tool categories across the studies (see Additional file 2: Appendix S2). We additionally incorporated LMS and the devices used, due to their relevance and presence in some of these publications. 14.2% of the studies (*n* = 40) included the exploration of the type of devices used by the participants for teaching and learning, such as handheld device and internet access (e.g., Adnan & Anwar, 2020). However, in 20.6% of the studies (*n* = 58) no specific technology, tools or devices were specified, often using a phrase such as ‘online learning’ to refer to all forms of technology used.

The most often mentioned type of tools were *synchronous collaboration tools*, especially video conferencing systems (e.g. Zoom, Teams, Google Meet, etc.) (51.8%, see Additional file 10: Appendix S10). Since many universities had to move from presence teaching to online teaching rapidly, many adopted video conference to replace the presence teaching traditional sessions. LMS were also popular in these publications (41.5%), which is in line with the use of the main institutional systems that the majority of universities already had and used to some extent (e.g. to upload materials). Other typologies of tools with important presence were *text-based tools* (especially text-based communication such as email or instant messaging, 31.9%) and *multimodal production tools* (34.8%), particularly the use of teachers’ pre-recorded videos.

Interestingly, the specific use of *assessment tools* was only 22.3%, which was lower than that found in a review of 242 pre-pandemic higher education studies (Bond et al., 2020), at 26.8%. Given the need to switch to online forms of assessment during the pandemic, this low number was quite surprising. Studies that did explicitly discuss the use of online assessment tools mentioned student concerns around not completing tests in time and online quizzes being inflexible with answers (Dietrich et al., 2020), internet connections dropping out during tests and affecting completion (Means & Neisler, 2020), the use of ‘just in time’ quizzes delivered to students’ mobile devices (Chen et al., 2021), and educator concerns over using online proctoring services (Cutri et al., 2020).

**Fig. 6:**
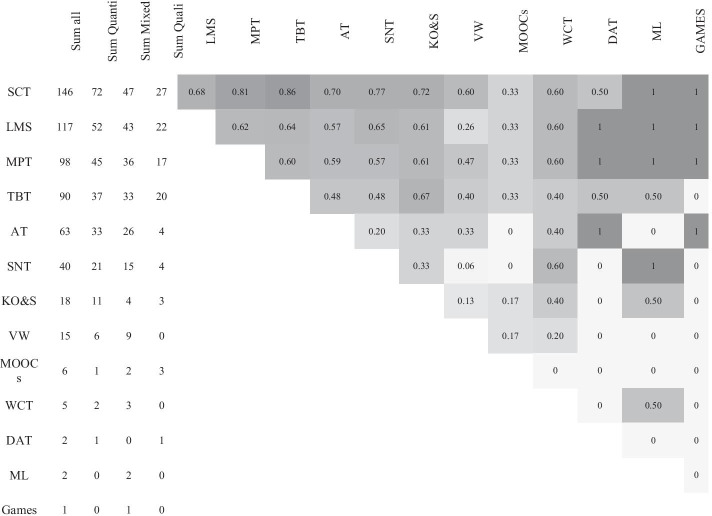
Co-occurrence of tools across the sample (*n* = 282). Note: Quanti = Quantitative, Quali = Qualitative, SCT = synchronous collaboration tools, LMS = learning management system, MPT = multimodal production tools, TBT = text-based tools, AT = assessment tools, SNT = social networking tools, KO&S = knowledge organisation & sharing tools, VW = virtual worlds, WCT = website creation tools, DAT = data analysis tools, ML = mobile learning

In order to provide further insight into the types of technology used during the pandemic, a tool co-occurrence was conducted (see Fig. 6). *Synchronous collaboration tools* was most often used with other technology types, being combined with *text-based tools* in 86% of all possible cases, with *multimodal production tools* in 81% of all possible cases, and with *social networking tools* in 77% of all possible cases. The increased use of video conferencing and pre-recorded videos during the pandemic becomes even further apparent, when the combination of tools are considered in comparison to pre-pandemic research. There was a much higher use of *synchronous collaboration tools* in combination with *text-based tools* in this review (86%), in comparison with 69% in a review of pre-pandemic higher education studies (Bond et al., 2020). Likewise, the combination of *synchronous collaboration tools* and *multimodal production tools* (81%), as well as the combination of *synchronous collaboration tools* and LMS (68%) was strikingly higher than that found in the review by Bond, Buntins et al. (2020b), with 56% and 38% respectively.

## Discussion and conclusion

This review mapped 282 studies conducted during the first ten months of the COVID-19 pandemic in 2020. The results from this mapping study are revealing in the sense that they allow a glimpse into a field of research that has been emerging heavily and quickly within a short period of time, but that continues to grow in parallel with the ongoing pandemic. Whilst the overall topic of emergency remote teaching is driven by the current COVID-19 situation, several results from this review are in line with pre-pandemic research in the field of educational technology.

The pandemic struck higher education unexpectedly, with swift decisions and actions enforced (ERT; Hodges et al., 2020). Ensuing research, as collated per this review, indicates that the scope of studies overwhelmingly resides in the *perceptions* of students of the switch to online teaching and learning—although perhaps not of all students, with a noticeable lack of consideration of vulnerable populations such as international and SEND students as part of the general student body found across the studies—and, to a lesser extent, on the perceptions of educators. That is, the opinions, experiences and perceptions of stakeholders were evaluated and considered, particularly through the use of surveys, but less so actual learning behaviour, grade differences or changes in study performance of students. This is not surprising, as this kind of research is easier to conduct—especially in the given circumstances—and is still informative of how students lived through the opening months of the pandemic. This finding is also in line with the fact that the majority of studies were carried out cross-sectionally, and employed descriptive statistics rather than more complex analyses. Thus, while teaching and learning were organised in a pragmatic manner, research was as well. This was particularly highlighted by the fact that only 10.6% of studies were grounded by theoretical frameworks, which is comparatively low in comparison with previous pre-pandemic research (Bond et al., 2020) and the wider field of educational technology (e.g., Castañeda & Selwyn, 2018; Hew et al., 2019). Framing research within a stronger theoretical basis, can assist with interpreting data (Kaliisa & Picard, 2017) and with identifying a “field’s disciplinary alignment” (Crook, 2019, p. 486). With the studies being set with a focus on the higher education context, aligning theories and research perspectives within the field of educational technology would be inconclusive were not the broader context of higher education research considered (e.g. Tight, 2018, 2020). With Tight (2020) showing that systematic reviews and meta-analyses within higher education research address the topic of course design in 289 out of 515 identified cases of overview works, the topic of teaching and learning is one of the most visibly researched one within this field. Operating more closely at the intersection of educational technology and higher education research, will be fruitful to add complementing points of view. As more time passes, it will be interesting to look out for changes in research currently being conducted. It is to be expected that after online learning and teaching has become more established within institutions, more profound and elaborate studies will follow. Indeed, whereas the goal of the present article was to provide an overview of the structure of research undertaken during the pandemic, further synthesis of the data in this review in the future will be guided by a bioecological model of student engagement (Bond & Bedenlier, 2019), through which more nuanced understandings of ERE can be derived.

The global geographical distribution of authors and participants’ country affiliations likewise mirrors previous findings (e.g., Bond et al., 2019), with a noticeable lack of research coming from the Global South and Oceanic countries. However, this should be considered within the specific context of the pandemic, given the variable numbers of infections in those locations in 2020, as well as the varying national responses to COVID-19 and measures employed to fight the spread of the pandemic. Also, this lack of research found in the review may also be related to two limitations of the study: research could be indexed in other databases that are not the ones analysed, or they could be published within their own languages, different from English and Spanish. Regarding the latter, we have to acknowledge that international databases mostly index journals that only accept submissions in English, and therefore, the number of papers written in that language—even considering that these may come from authors in non-English speaking countries—is much higher than other languages (Tight, 2019).

The results on geographical distribution in this study, point to questions that arise around the way that global academic publishing works that are not specifically pertinent to this study. Being more sensitive to global publishing structures, and framing results of descriptive studies like this one within the broader discourse on global academic publishing with its separation in centres and peripheries, is deemed important for ensuing research endeavours (e.g. Altbach, 2016; Marín & Zawacki-Richter, 2019; Mosbah-Natanson & Gingras, 2014). Using the example of the Latin American publishing context, Beigel (2021) finds that within the so-called periphery, regional centres emerge, which calls for closer inspection of publication patterns within regions. Furthermore, the increased number of open access journals is “a fruitful path to co-construction of knowledge” (Beigel, 2014, p. 619), nurturing the hope that a more even spread of publications across world regions is possible.

Other possible and pragmatic interpretations for the reduced research found from the Global South and Oceanic countries are that many institutions from some of those countries may have been relying on online education previously and, therefore, the situation was not new for them; or that may have had problems with Internet access or not able to undertake research due to the pandemic. On the other hand, although the pandemic struck globally, academic collaboration in research occurred mostly with domestic colleagues and in teams working with two or three authors. It can be assumed that researchers had – in their double-function as teacher and researcher – relatively easy access to students at their respective institution. In contrast, the conditions, semester timing and other factors, certainly varied from country to country (Bozkurt et al., 2020), and doing international comparative research might have been harder to realise; aspects that will be further explored in our future research, applying specifically to the consideration of knowledge construction and dissemination as a distinct topic in itself, and a meta-perspective to the results of this review.

As found in K-12 research conducted during the early stages of the pandemic (Bond, 2020), approximately one third of research omitted important study design information. This ranged from information about student participants’ study levels, to the discipline/ subjects under investigation, whether ‘first year’ or ‘introductory’ referred to undergraduate or postgraduate students, to information about the exact technology being employed within courses. The concept of online or digital learning can vary an extraordinary amount between contexts and studies, and in order for readers to understand and consider whether a study is applicable to their own situation, full study design details must be provided (Bond et al., 2020; Slavin, 2008).

The large number of studies published open access, mirrors the relevance of initiatives such as the Open Covid Pledge for Research in Education (https://www.alt.ac.uk/about-alt/what-we-do/open-covid-pledge-education), in spite of the forecast analysis on general overall research on COVID-19 literature being more restrictive (Torres-Salinas et al., 2020). With the broad range of publication outlets, ranging from educational technology to discipline-specific journals, as well as pre-print and academic repositories, it is again evident how forceful COVID-19 has impacted higher education and the actors involved.

It can be surmised that the educational technology most often employed—*synchronous collaboration tools*, and especially video conferencing—is because of the fact that teachers and students had the urge to re-create communication and interaction situations that are found during in-person lessons on campus (Giovannella, 2021), with a high potential of simulating face-to-face communication. With video conferencing being around for thirty years (Bonk, 2020), it has certainly experienced a surge in interest and broad application, entailing other questions related to its use (Bedenlier et al., 2020; Castelli & Sarvary, 2021). The use of video conferencing and the reliance on synchronous courses/meetings as part of online teaching and learning does, however, also indicate that at the outset of the first semester, larger numbers of teachers can be assumed to have not yet had extensive experience in providing other formats of online learning that make more use of the temporal flexibility inherent to online formats. What is also evident in the reviewed studies, is that another educational technology tool that was used frequently—the learning management system—whilst not surprising, is interesting in the sense that it is potentially used more often for teaching and learning purposes, and not solely as a file repository (Brady & O’Reilly, 2020). Only a small number of studies referred to having employed more advanced educational technology, such as virtual worlds, simulations, e-portfolios or similar, as well as a smaller than expected focus on online and alternative methods of assessment. Given the circumstances, this is perhaps not surprising, and also aligns with pre-pandemic use of educational technology (e.g., Bond et al., 2020b). This does beg the question, however, of whether the expectations of COVID-19 as a catalyst for educational change have been—or will be—realised (Zhao, 2020), and that there is a further need for greater research into how the digital transformation of higher education should be developed (García-Peñalvo, 2021).

The next stage of this research is to conduct a further iteration of the search strategy and data extraction, in order to bring the review up to date. Researchers are warmly invited to contact the review team with suggestions of studies for inclusion, as a publicly available database will be created using EPPI-Reviewer (see Bond et al., 2021), which will be searchable and filterable, and contain several interactive evidence gap maps, which researchers, policy makers and educators can explore, download and use to inform policy and practice. More extensive data extraction will also occur, in order to synthesise results and gain deeper knowledge of which technology worked well and why.

## Supplementary Information

**Additional file 1: Appendix S1.** Tabulated list of included studies (*n* = 282).

**Additional file 2: Appendix S2.** Educational technology tool typology based on Bower (2016).

**Additional file 3: Appendix S3.** List of publications (*n* = 155).

**Additional file 4: Appendix S4.** Scope of participant focus.

**Additional file 5: Appendix S5.** Number of participants per study (n = 282).

**Additional file 6: Appendix S6.** Crosstabulation of disciplines (*n* = 282).

**Additional file 7: Appendix S7.** Data collection methods (*n* = 282).

**Additional file 8: Appendix S8.** Data analysis methods (n = 282)

**Additional file 9: Appendix S9.** Terminology used to describe teaching and learning during the pandemic (n = 282)

**Additional file 10: Appendix S10.** Technology used based on Bower’s (2016) typology (n = 282)

## Data Availability

The datasets generated and analysed during the current study are available on ResearchGate, as well as on the EPPI-Centre website.

## Footnotes

1 https://www.researchgate.net/community/COVID-19.

2 https://www.researchgate.net/project/Mapping-the-emerging-field-of-research-on-emergency-remote-teaching-in-higher-education-due-to-COVID-19-Implications-for-education-research-and-practice.

3 For more information about EPPI-Mapper and creating interactive evidence gap maps, see https://eppi.ioe.ac.uk/cms/Default.aspx?tabid=3790.

4 https://info.leximancer.com/.

5 https://www.fachportal-paedagogik.de/literatur/produkte/fis_bildung/fis_bildung.html.

6 https://beallslist.net/standalone-journals/#update.

7 See https://pubs.acs.org/doi/10.1021/acs.jchemed.0c00378 for more information.
