# Supplementary material for: Emergency remote teaching in higher education: mapping the first global online semester
Source: Int J Educ Technol High Educ. 2021 Aug 30;18(1):50. doi: 10.1186/s41239-021-00282-x (PMC8403509; doi:10.1186/s41239-021-00282-x)
Supplement: Supplementary file 1 — Additional file 1: Appendix S1. Tabulated list of included studies (n = 282). [file 41239_2021_282_MOESM1_ESM.docx]

# Appendix A – Tabulated list of included studies (*n* = 282)

| **Author** | **Continent** | **Country** | **Participants** | **# Participants** | **Study level** | **Discipline** | **Technology used** |
| --- | --- | --- | --- | --- | --- | --- | --- |
| [Abbasi et al.](https://doi.org/10.12669/pjms.36.COVID19-S4.2766) | Asia | Pakistan | S | 382 | UG | H&W | D |
| [Abdulrahim & Mabrouk](http://www.asianjde.org/ojs/index.php/AsianJDE/article/view/468) | Middle East | Saudi Arabia | S, T | 250 | UG, PG | A&H, NSM, H&W, Eng | N/S |
| [Aboagye, Yawson & Appiah](https://doi.org/10.37256/ser.122020422) | Africa | Ghana | S | 141 | N/S | N/S | N/S |
| [Aboagye](https://doi.org/10.37256/ser.212021545) | Africa | Ghana | T | 63 | - | Edu | LMS |
| [Adnan & Anwar](http://files.eric.ed.gov/fulltext/ED606496.pdf) | Asia | Pakistan | S | 126 | UG, PG | N/S | D |
| [Agoestyowati](https://ojs.stiami.ac.id/index.php/ABIWARA/article/view/1049) | Asia | Indonesia | S | 5 | N/S | N/S | SCT, TBT |
| [Agormedah et al.](https://doi.org/10.31681/jetol.726441) | Africa | Ghana | S | 467 | UG | N/S | LMS |
| [Ahmed et al.](https://doi.org/10.1186/S12909-020-02311-1) | Global | Global | T, DM | 79 | - | H&W | N/S |
| [Akande et al.](https://doi.org/10.1016/j.dib.2020.105926) | Africa | Nigeria | S | 850 | N/S | N/S | SNT |
| [Al-Maroof et al.](https://doi.org/10.1080/10494820.2020.1830121) | Middle East | UAE | S | 450 | UG, PG | A&H, B, ICT | SCT |
| [Al-Nofaie](https://doi.org/10.24093/awej/vol11no3.1) | Middle East | Saudi Arabia | S | 25 | UG | A&H | LMS, SCT, TBT, MPT, AT |
| [Al-Okaily et al.](https://doi.org/10.18510/HSSR.2020.8483) | Middle East | Jordan | S | 587 | UG | Edu, A&H, NSM, Eng, B, ICT | N/S |
| [Al-Tammemi, Akour & Alfalah](https://doi.org/10.3389/fpsyg.2020.562213) | Middle East | Jordan | S | 701 | UG, PG | N/S | N/S |
| [Al-Taweel et al.](https://doi.org/10.1111/eje.12589) | Middle East | Iraq | S | 832 | UG | H&W | MPT, SNT, LMS |
| [Aleksandra et al.](https://www.researchgate.net/publication/345940913_THE_DIFFERENCES_IN_STUDENTS'_ATTITUDES_ABOUT_ONLINE_TEACHING_DURING_COVID-19_PANDEMIC) | Europe | Serbia | S | 129 | UG | Edu | LMS |
| [Allam et al.](https://doi.org/10.6007/IJARBSS/V10-I5/7236) | Asia | Malaysia | S | 631 | UG | A&H | D |
| [Almaiah, Al-Khasawneh & Althunibat](https://doi.org/10.1007/s10639-020-10219-y) | Middle East | Saudi Arabia, Jordan | S, T, SS, PM | 67 | N/S | N/S | N/S |
| [Almuraqab](https://www.ncbi.nlm.nih.gov/pmc/articles/PMC7366799/) | Middle East | UAE | S | 133 | UG, PG | Eng, B, ICT | D |
| [Alqahtani & Rajkhan](https://doi.org/10.3390/educsci10090216) | Middle East | Saudi Arabia | SS | 69 | - | Edu, A&H, NSM, H&W, Eng, B, SSci | N/S |
| [Alqudah et al.](https://doi.org/10.1016/j.amsu.2020.09.014) | Middle East | Jordan | T | 22 | - | H&W | SCT, TBT, MPT, SNT, LMS |
| [Alqurshi](https://doi.org/10.1016/j.jsps.2020.07.008) | Middle East | Saudi Arabia | S, T | 777 | UG | H&W | AT |
| [Alsadoon & Turkestani](https://doi.org/10.18662/rrem/12.1sup2/240) | Middle East | Saudi Arabia | T | 11 | - | N/S | SCT, TBT, LMS |
| [Alshehri et al.](https://doi.org/10.4236/ce.2020.117071) | Middle East | Saudi Arabia | T | 122 | - | A&H, B, ICT | SCT, LMS |
| [Altinay et al.](https://doi.org/10.18662/rrem/12.1sup2/241) | Europe | Cyprus | T, DM | 15 | - | N/S | N/S |
| [Alturise](https://dx.doi.org/10.14569/IJACSA.2020.0110512) | Middle East | Saudi Arabia | S, T | 661 | N/S | N/S | LMS |
| [Alvarez](https://doi.org/10.5281/zenodo.3881529) | Asia | Philippines | S | 5 | UG | N/S | LMS, D |
| [Amaya, Cantú & Marreros](https://dialnet.unirioja.es/servlet/articulo?codigo=7710787) | S & C America | Mexico | T | 87 | - | N/S | N/S |
| [Amin & Sundari](https://doi.org/10.24815/siele.v7i2.16929) | Asia | Indonesia | S | 140 | UG | A&H | SNT |
| [Amir et al.](https://doi.org/10.1186/s12909-020-02312-0) | Asia | Indonesia | S | 301 | UG | H&W | SCT, MPT, LMS |
| [Anggraeni & Pentury](https://doi.org/10.26858/RETORIKA.V13I2.13538) | Asia | Indonesia | S | 84 | UG | A&H | SCT, TBT, LMS |
| [Anwar, Khan & Sultan](http://www.gujr.com.pk/index.php/GUJR/article/view/1206) | Asia | Pakistan | S | 450 | N/S | Edu, A&H, NSM, SSci, ICT | N/S |
| [Anzovino et al.](https://doi.org/10.1021/acs.jchemed.0c00766) | North America | United States | S | 1,095 | UG | NSM | MPT, AT, LMS |
| [Area-Moreira et al.](http://uajournals.com/ojs/index.php/campusvirtuales/article/view/733) | Europe | Spain | S | 51 | UG | Edu | N/S |
| [Aristovnik et al.](https://doi.org/10.3390/su12208438) | Global | Global | S | 30,383 | UG, PG | A&H, NSM, SSci | SCT, TBT, MPT, D |
| [Armstrong-Mensah et al.](https://doi.org/10.3389/fpubh.2020.576227) | North America | United States | S | 184 | UG, PG | H&W | TBT, MPT, AT, D |
| [Arroyo-Vázquez & Gómez-Hernández](https://doi.org/10.3145/epi.2020.jul.04) | Europe | Spain | SS | 14 | - | SSci | SCT |
| [Aucejo et al.](https://doi.org/10.1016/j.jpubeco.2020.104271) | North America | United States | S | 1,446 | UG | N/S | N/S |
| [Azis & Fatimah](https://doi.org/10.47577/TSSJ.V9I1.1033) | Asia | Indonesia | S | 65 | UG, PG | B | LMS |
| [Azlan et al.](https://doi.org/10.1016/J.EJMP.2020.10.002) | Asia | Malaysia | S | 11 | PG | H&W | KOT, SCT, TBT, MPT, AT, LMS |
| [Baber](https://doi.org/10.20448/journal.509.2020.73.285.292) | Asia | India, South Korea | S | 100 | UG | N/S | N/S |
| [Baloran](https://doi.org/10.1080/15325024.2020.1769300) | Asia | Philippines | S | 530 | N/S | N/S | N/S |
| [Barkanian](https://www.arabianjbmr.com/pdfs/Arabian%20Journal%20of%20Business%20and%20Management%20Review%20(Kuwait%20Chapter)_KD_VOL_9_2_2020/1_ajbmrkd_09022020.pdf) | Middle East | Lebanon | S, T | 170 | N/S | N/S | D |
| [Barova & Mileva](https://azbuki.bg/wp-content/uploads/2020/09/NSA-Iva-Barova-Eleonora-Mileva.pdf) | Europe | Bulgaria | S | 116 | UG | H&W | SCT |
| [Barra et al.](https://doi.org/10.3390/su12187451) | Europe | Spain | S | 85 | UG | Eng | AT |
| [Barrera et al.](http://www.risti.xyz/issues/ristie31.pdf) | S & C America | Colombia | S, T, SS | 2,432 | N/S | Edu, Eng, B, SSci, ICT, Other | N/S |

Note: UAE = United Arab Emirates, S & C America = South & Central America, T = Teachers/Instructors, S = Students, DM = Department Managers, SS = Support Staff, PM = Policy makers, UG = Undergraduate, PG = Postgraduate, H&W = Health & Welfare, A&H = Arts & Humanities, NSM = Natural Science, Mathematics & Statistics, Eng = Engineering, Manufacturing & Construction, Edu = Education, B = Business, Administration & Law, N/S = Not specified, SSci = Social Sciences, Journalism & Information, LMS = Learning Management System, SNT = Social networking tools, SCT = Synchronous collaboration tools, TBT = Text-based tools, MPT = Multimedia production tools, AT = Assessment tools, KOT = Knowledge organization & sharing tools, D = Devices used

| **Author** | **Continent** | **Country** | **Participants** | **# Participants** | **Study level** | **Discipline** | **Technology used** |
| --- | --- | --- | --- | --- | --- | --- | --- |
| [Bartz](https://doi.org/10.3390/educsci10090223) | Europe | Germany | S | 45 | N/S | N/S | SCT, MPT |
| [Başal & Eryılmaz](https://doi.org/10.1080/02607476.2020.1841555) | Middle East | Turkey | S | 92 | UG | Edu | TBT, MPT |
| [Batajoo et al.](https://journal.kistmcth.edu.np/index.php/JKISTMC/article/view/77) | Asia | Nepal | S | 88 | UG | H&W | SCT |
| [Baticulon et al.](https://doi.org/10.1101/2020.07.16.20155747) | Asia | Philippines | S | 3,670 | N/S | H&W | D |
| [Bautista et al.](https://doi.org/10.1080/13561820.2020.1807920) | North America | United States | S | 5 | UG | H&W | SCT, TBT, MPT |
| [Bawaneh](https://www.researchgate.net/publication/346570473_The_Satisfaction_Level_of_Undergraduate_Science_Students_towards_Using_e-_Learning_and_Virtual_Classes_in_Exceptional_Condition_COVID-19_Crisis) | Middle East | Saudi Arabia | S | 116 | UG | NSM | N/S |
| [Berkova & Nemec](https://doi.org/10.3390/SYM12091577) | Europe | Czech Republic | S | 41 | N/S | NSM | MPT, AT |
| [Besser, Lotem & Zeigler-Hill](https://doi.org/10.1016/j.jvoice.2020.05.028) | Middle East | Israel | T | 313 | - | N/S | D |
| [Bhargava](https://www.jrmds.in/articles/online-classes-for-medical-students-during-covid19-pandemic-through-the-eyes-of-the-teaching-faculty-55416.html) | Asia | India | T | 40 | - | H&W | SCT |
| [Blizak et al.](https://doi.org/10.1021/acs.jchemed.0c00668) | Africa | Algeria | S | 380 | UG, PG | NSM | SCT, TBT, SNT, LMS |
| [Bogdandy, Tamas & Toth](https://ieeexplore.ieee.org/document/9237840) | Europe | Hungary | S | 60 | N/S | Eng, ICT | N/S |
| [Cameron-Sanderford et al.](https://doi.org/10.3389/FEDUC.2020.583881) | North America | United States | T | 83 | - | N/S | LMS |
| [Cano et al.](http://uajournals.com/ojs/index.php/campusvirtuales/article/viewFile/734/412) | Europe, S & C America | Argentina, Spain, Mexico, Colombia, Chile, Portugal | S, T | 175 | N/S | N/S | SCT, TBT, AT, SNT, LMS |
| [Capone et al.](https://doi.org/10.3390/su12177039) | Europe | Italy | S | 1,124 | UG, PG | N/S | N/S |
| [Carrascosa et al.](https://doi.org/10.1590/1806-9282.66.6.812) | S & C America | Brazil | S | 317 | N/S | H&W | N/S |
| [Casillas et al.](https://doi.org/10.48102/rlee.2020.50.ESPECIAL.97) | S & C America | Multiple | T | 1,310 | - | N/S | N/S |
| [Chen, Jiao & Hu](https://doi.org/10.4018/IJDET.20210101.oa1) | Asia | China | S | 60 | UG | A&H | SCT, AT, D |
| [Choi et al.](https://doi.org/10.1186/s12909-020-02117-1) | Europe | United Kingdom | S | 440 | N/S | H&W | AT |
| [Cigerci](http://www.ijtase.net/ojs/index.php/IOJPE/article/view/1028) | Middle East | Turkey | S | 15 | UG | Edu | KOS, SCT, MPT, LMS |
| [Cirillo et al.](https://learntechlib.org/primary/p/216305/) | North America | United States | S, T | 14 | N/S | Edu | SCT |
| [Co & Chu](https://doi.org/10.1111/1744-1633.12436) | Asia | Hong Kong | S | 30 | UG | H&W | SCT, MPT |
| [Cohen & Davidovitch](https://doi.org/10.5539/jel.v9n5p15) | Middle East | Israel | S | 1,400 | UG, PG | N/S | MPT |
| [Craig et al.](https://doi.org/10.1108/ILS-04-2020-0105) | North America | United States | S | 49 | UG | A&H | KOS |
| [Crick, Knight et al.](https://doi.org/10.1145/3416465.3416472) | Europe | United Kingdom | T | 1,148 | - | Eng | N/S |
| [Crucho et al.](https://doi.org/10.1021/acs.jchemed.0c00693) | Europe | Portugal | S | 60 | UG | NSM | SCT, TBT, MPT, AT, VW, LMS |
| [Cuschieri & Calleja Agius](https://doi.org/10.1002/ASE.2020) | Europe | Malta | S | 172 | UG | H&W | MPT |
| [Cutri, Mena & Whiting](https://doi.org/10.1080/02619768.2020.1815702) | Europe, North America | United States, Mexico, Russia | T | 30 | - | Edu | AT |
| [Danjou](https://doi.org/10.1021/acs.jchemed.0c00485) | Europe | France | S | 20 | UG | NSM | MPT, SNT, D |
| [Dasheva, Andonov & Doncheva](https://www.researchgate.net/publication/344375766_MASTER%27S_PROGRAM_HIGH_PERFORMANCE_SPORT_E-LEARNING_DURING_COVID-19_PANDEMIC) | Europe | Bulgaria | T | 92 | - | H&W | SCT, TBT, LMS |
| [Dickson-Karn](https://doi.org/10.1021/acs.jchemed.0c00578) | North America | United States | S | 11 | UG | NSM | TBT, AT |
| [Dietrich et al.](https://doi.org/10.1021/acs.jchemed.0c00717) | Europe | France | S, T | 103 | UG | NSM | SCT, MPT, AT, LMS |
| [Dingwall](https://doi.org/10.1021/acs.jchemed.0c00810) | North America | United States | S | 304 | UG | NSM | SCT, TBT, MPT, LMS |
| [Dinh & Nguyen](https://doi.org/10.1080/02615479.2020.1823365) | Asia | Vietnam | S | 186 | UG | SSci | SCT, TBT, D |
| [Dost et al.](https://doi.org/10.1136/BMJOPEN-2020-042378) | Europe | United Kingdom | S | 2,721 | UG, PG | H&W | SCT, MPT, AT, G, LMS |
| [Driessen et al.](https://doi.org/10.1002/ece3.6741) | North America | United States | S | 330 | UG | NSM | MPT, AT, LMS |
| [Dushkevych et al.](https://doi.org/10.18662/rrem/12.1sup2/248) | Europe | Ukraine | S | 83 | N/S | A&H | SCT, TBT, LMS |
| [Dwidienawati et al.](https://doi.org/10.30534/ijatcse/2020/93922020) | Asia | Indonesia | S | 153 | UG | N/S | N/S |
| [Edelhauser & Lupu-Dima](https://doi.org/10.3390/su12135438) | Europe | Romania | S | 209 | UG, PG | NSM, Eng | SCT, AT, SNT, LMS, D |
| [Elledge et al.](https://doi.org/10.1016/j.bjoms.2020.07.030) | Europe | United Kingdom | S | 95 | PG | H&W | SCT |
| [Ellis, Steadman & Mao](https://doi.org/10.1080/02619768.2020.1821186) | Asia, Europe, N. America, Oceania | Multiple | DM | 10 | - | Edu | SCT, MOOC |
| [Elsalem et al](https://doi.org/10.1016/J.AMSU.2020.10.058). | Middle East | Jordan | S | 1,019 | N/S | H&W | AT |
| [Elumalai et al.](https://doi.org/10.28945/4628) | Middle East, Asia | India, Saudi Arabia | S | 784 | UG, PG | N/S | LMS |

Note: UAE = United Arab Emirates, S & C America = South & Central America, N. America = North America, T = Teachers/Instructors, S = Students, DM = Department Managers, A = Administrators, SS = Support Staff, UG = Undergraduate, PG = Postgraduate, H&W = Health & Welfare, A&H = Arts & Humanities, NSM = Natural Science, Mathematics & Statistics, Eng = Engineering, Manufacturing & Construction, Edu = Education, B = Business, Administration & Law, N/S = Not specified, SSci = Social Sciences, Journalism & Information, LMS = Learning Management System, SNT = Social networking tools, SCT = Synchronous collaboration tools, TBT = Text-based tools, MPT = Multimedia production tools, AT = Assessment tools, KOT = Knowledge organization & sharing tools, VW = Virtual Worlds, D = Devices used

| **Author** | **Continent** | **Country** | **Participants** | **# Participants** | **Study level** | **Discipline** | **Technology used** |
| --- | --- | --- | --- | --- | --- | --- | --- |
| [Eurboonyanun et al.](https://doi.org/10.1016/J.JSURG.2020.08.046) | Asia | Thailand | S | 47 | UG | H&W | AT |
| [Fatani](https://doi.org/10.1186/s12909-020-02310-2) | Middle East | Saudi Arabia | S | 162 | UG | H&W | SCT |
| [Fawaz & Samaha](https://doi.org/10.1111/nuf.12521) | Middle East | Lebanon | S | 520 | UG | N/S | N/S |
| [Fey, Theus & Ramirez](https://doi.org/10.1002/ece3.6916) | North America | United States | S | 20 | UG | NSM | SCT, SNT |
| [Fox, Werth et al.](https://arxiv.org/pdf/2007.01271.pdf) | North America | United States | T | 106 | - | NSM | VW |
| [Fox, Bryant et al.](https://www.everylearnereverywhere.org/resources/time-for-class-covid-19-edition/) | North America | United States | T | 4,798 | - | N/S | MPT, SCT, TBT, AT, LMS |
| [Fung & Lam](https://doi.org/10.1021/acs.jchemed.0c00590) | Asia | Singapore | S | 154 | UG | NSM | SCT, MPT, SNT |
| [Gao, Lloyd & Kim](https://doi.org/10.1021/acs.jchemed.0c00634) | North America | United States | S | 16 | UG | NSM | VW |
| [Gao & Zhang](https://doi.org/10.3389/fpsyg.2020.549653) | Asia | China | T | 3 | - | A&H | KOS, TBT, LMS, MOOC |
| [Gaur et al.](https://doi.org/10.18203/2394-6040.IJCMPH20203892) | Asia | India | S | 394 | UG | H&W | N/S |
| [Gelles et al.](https://doi.org/10.3390/EDUCSCI10110304) | North America | United States | S | 11 | UG | Eng | SCT, MPT, LMS |
| [Getova, Mileva & Angelova-Igova](https://azbuki.bg/wp-content/uploads/2020/09/NSA-Antoaneta-Getova-Eleonora-mileva-Boryana-Angelova-Igova.pdf) | Europe | Bulgaria | S | 427 | N/S | Edu, A&H, NSM, H&W | KOS, SCT, TBT, LMS |
| [Ghounane](https://doi.org/10.24093/awej/vol11no3.2) | Africa | Algeria | S, T | 98 | PG | A&H | MPT, LMS, SCT, TBT, SNT |
| [Gill, Brito & Quintero](http://scielo.sld.cu/scielo.php?script=sci_arttext&pid=S1990-86442020000500325&lng=pt&nrm=iso) | S & C America | Ecuador | S | 627 | UG | Edu | SCT, TBT, LMS |
| [Gillis & Krull](https://doi.org/10.1177/0092055X20954263) | North America | United States | S | 66 | UG | SSci | SCT, TBT, MPT, LMS |
| [Gonzalez et al.](https://doi.org/10.1371/journal.%20pone.0239490) | Europe | Spain | S | 458 | N/S | H&W | N/S |
| [González-Calvo et al.](https://doi.org/10.17583/rimcis.2020.5783) | Europe | Spain | S | 8 | UG | Edu | N/S |
| [Guangul et al.](https://doi.org/10.1007/s11092-020-09340-w) | Middle East | Oman | T | 50 | - | N/S | AT, LMS, WCT |
| [Guban-Caisado](https://doi.org/10.37237/110305) | Asia | Philippines | S | 10 | N/S | A&H | MPT, LMS, D |
| [Guo](https://doi.org/10.1088/1361-6552/ABA1C5) | North America | United States | S | 20 | UG | NSM | SCT, VW |
| [Gupta, Pandey & Anshu](https://doi.org/10.3329/bjms.v19i0.47831) | Asia | India | S | 243 | UG | H&W | SCT, AT |
| [Gupta et al.](https://doi.org/10.3126/OJN.V10I2.31064) | Asia | Nepal | S | 769 | UG | H&W | SCT |
| [Gyampoh et al.](http://www.netjournals.org/pdf/AERJ/2020/3/20-088.pdf) | Africa | Ghana | T | 24 | - | Edu | SCT, TBT, D |
| [Hadar et al.](https://doi.org/10.1080/02619768.2020.1807513) | Middle East | Israel | S, T | 78 | PG | Edu | N/S |
| [Hadi, Andrian & Hidayat](https://www.iratde.com/index.php/jtde/article/view/1453) | Asia | Indonesia | S | 167 | N/S | N/S | TBT, LMS |
| [Händel et al.](http://dx.doi.org/10.1080/15391523.2020.1846147) | Europe | Germany | S | 1,826 | UG, PG | A&H, NSM, H&W, Eng, B, SSci | D |
| [Händel, Bedenlier et al.](https://doi.org/10.31234/osf.io/5ngm9) | Europe | Germany | S | 5,563 | UG, PG | A&H, Edu, NSM, H&W, Eng, B | D |
| [Hashim et al.](https://www.bibliomed.org/mnsfulltext/27/27-1586730807.pdf?1610375489) | Asia | Pakistan | S | 239 | UG | H&W | N/S |
| [Heyang & Martin](https://doi.org/10.1080/14647893.2020.1780206) | Asia, Europe | China, Norway | T | 2 | - | A&H | SCT |
| [Howitz et al.](https://doi.org/10.26434/chemrxiv.12605207.v2) | North America | United States | S, T | 1431 | UG | NSM | SCT, TBT, MPT, AT, LMS, D |
| [Howitz, Guaglianone & King](https://doi.org/10.1021/acs.jchemed.0c00809) | North America | United States | S | 260 | UG | NSM | SCT, TBT, MPT, AT, LMS, D |
| [Huang](https://doi.org/10.1021/acs.jchemed.0c00671) | Asia | China | S, T | 488 | UG | NSM | MPT, VW, LMS, MOOC, D |
| [Hwang](https://doi.org/10.1021/acs.jchemed.0c00808) | North America | United States | S | 117 | UG | NSM | AT, SNT, LMS |
| [Ibrahim, Luzinge & Kapanda](https://jl4d.org/index.php/ejl4d/article/view/448) | Africa | Tanzania | S, T | 1,596 | UG, PG | H&W | SCT, MPT, LMS, D |
| [Iqbal et al.](https://doi.org/10.5455/aim.2020.28.94-97) | Middle East | Saudi Arabia | S | 203 | UG | H&W | TBT |
| [Irfan et al.](https://doi.org/10.22460/INFINITY.V9I2.P147-158) | Asia | Indonesia | T | 26 | - | Edu | SCT, MPT, LMS |
| [Ivanović, Gajević & Gajić](https://azbuki.bg/wp-content/uploads/2020/09/NSA-Jelena-Ivanovic-Aco-Gajevic.pdf) | Europe | Serbia | S | 27 | UG | H&W | SCT, LMS |
| [Jadhav et al.](http://ierj.in/journal/index.php/ierj/article/view/2154) | Asia | India | S | 373 | UG, PG | NSM, H&W, Eng | LMS |
| [Jankowski](https://www.learningoutcomesassessment.org/wp-content/uploads/2020/08/2020-COVID-Survey.pdf) | Africa, Asia, Europe, N. America, Middle East, S & C America | Multiple | T, DM | 813 | - | N/S | N/S |
| [Jeffery & Bauer](https://doi.org/10.1021/acs.jchemed.0c00736) | North America | United States | S | 208 | UG | NSM | SCT, MPT, LMS |
| [Jiménez-Rodríguez & Arrogante](https://doi.org/10.3390/HEALTHCARE8030280) | Europe | Spain | S | 93 | UG | H&W | VW |
| [Johnson, Veletsianos & Seaman](https://doi.org/10.24059/olj.v24i2.2285) | North America | United States | T, DM | 897 | - | N/S | SCT, TBT, MPT, SNT, LMS |
| [Johnson et al.](https://doi.org/10.1021/acs.jchemed.0c00728) | North America | United States | S | 38 | UG | NSM | SCT, TBT, MPT, AT, VW, LMS |
| [Jones, Vidal & Taylor](https://doi.org/10.1080/13561820.2020.1801614) | North America | United States | S | 587 | UG, PG | H&W | TBT, LMS |

Note: UAE = United Arab Emirates, S & C America = South & Central America, T = Teachers/Instructors, S = Students, DM = Department Managers, A = Administrators, SS = Support Staff, UG = Undergraduate, PG = Postgraduate, H&W = Health & Welfare, A&H = Arts & Humanities, NSM = Natural Science, Mathematics & Statistics, Eng = Engineering, Manufacturing & Construction, Edu = Education, B = Business, Administration & Law, N/S = Not specified, SSci = Social Sciences, Journalism & Information, Ag = Agriculture, Forestry, Fisheries & Veterinary, LMS = Learning Management System, SNT = Social networking tools, SCT = Synchronous collaboration tools, TBT = Text-based tools, MPT = Multimedia production tools, AT = Assessment tools, KOT = Knowledge organization & sharing tools, WCT = Website creation tools, D = Devices used

| **Author** | **Continent** | **Country** | **Participants** | **# Participants** | **Study level** | **Discipline** | **Technology used** |
| --- | --- | --- | --- | --- | --- | --- | --- |
| [Joshi, Vinay & Bhaskar](https://doi.org/10.1108/ITSE-06-2020-0087) | Asia | India | T | 19 | - | N/S | KOS, SCT, TBT, MPT, AT, D, LMS |
| [Kabanova et al.](https://doi.org/10.46925/rdluz.31.35) | Europe | Russia | S | 100 | UG | B | N/S |
| [Kamal et al.](https://doi.org/10.14569/IJACSA.2020.0110628) | Asia | Malaysia | S | 354 | N/S | NSM | AT |
| [Kapasia et al.](https://doi.org/10.1016/j.childyouth.2020.105194) | Asia | India | S | 232 | UG, PG | A&H, NSM, B | SCT, TBT, MPT, LMS |
| [Karalis & Raikou](http://dx.doi.org/10.6007/IJARBSS/v10-i5/7219) | Europe | Greece | S | 103 | UG | Edu | SCT, TBT |
| [Karatas & Tuncer](https://doi.org/10.3390/su12198188) | Middle East | Turkey | S | 118 | UG | Edu | MPT, LMS |
| [Kedraka & Kaltsidis](https://doi.org/10.46827/EJES.V7I8.3176) | Europe | Greece | S | 75 | UG | NSM | SCT, TBT |
| [Khalaf et al.](https://doi.org/10.1080/10872981.2020.1826861) | Middle East | UAE | S, T | 94 | UG | H&W | SCT, AT, D, LMS |
| [Kidd & Murray](https://doi.org/10.1080/02619768.2020.1820480) | Europe | United Kingdom | T | 14 | - | Edu | SCT, MPT |
| [Kikut](http://www.kerwa.ucr.ac.cr/handle/10669/81216) | S & C America | Costa Rica | S | 14,918 | UG | Edu, A&H, NSM, H&W, Eng, B, SSci | SCT, TBT, MPT, LMS, D |
| [Kim et al.](https://doi.org/10.3390/su12208433) | North America | United States | S | 240 | N/S | N/S | KOT, TBT, LMS |
| [Kirschner, Mohammed & Weiner](https://web.wpi.edu/Pubs/E-project/Available/E-project-071020-170937/unrestricted/CovidEdu_IQP_Report_E20.pdf) | North America | United States | S | 273 | N/S | Eng | SCT, MPT |
| [Klegeris](https://doi.org/10.1080/0309877X.2020.1826036) | North America | Canada | S | 89 | UG | NSM | KOS, TBT, MPT, SNT, LMS |
| [Klein et al.](https://arxiv.org/abs/2010.05622) | Europe | Germany, Austria, Croatia | S | 578 | UG, PG | NSM | SCT, TBT, MPT, D |
| [Knudson](https://doi.org/10.1080/14763141.2020.1810306) | North America | United States | S | 74 | N/S | H&W | TBT, MPT, AT, LMS |
| [Kockmann](https://doi.org/10.1002/cite.202000206) | Europe | Germany | S | 112 | UG, PG | N/S | N/S |
| [Kolar, Turcinovic & Bojanjac](https://doi.org/10.1109/ELMAR49956.2020.9219045) | Europe | Croatia | S | 819 | N/S | Eng | KOS, SCT, LMS, MPT |
| [Kollalpitiya, Partigianoni & Adsmond](https://doi.org/10.1021/acs.jchemed.0c00772) | North America | United States | S | 146 | UG | NSM | SCT, TBT |
| [Koretsky](https://doi.org/10.1021/acs.jchemed.0c00711) | North America | United States | S | 163 | UG | NSM | SCT, TBT, MPT, AT, VW |
| [Kostikova et al.](https://doi.org/10.20511/pyr2020.v8nSPE3.707) | Europe | Russia | S, T | N/S | UG, PG | N/S | SCT, MPT, SNT, LMS |
| [Krishnapatria](https://doi.org/10.35706/ELTINFC.V3I1.3694) | Asia | Indonesia | S, T | 62 | N/S | A&H | SCT, SNT, LMS |
| [Kusumawati](https://doi.org/10.26858/ijole.v4i2.14745) | Asia | Indonesia | S | 214 | UG | Eng | KOS, SCT, TBT, MPT, SNT, LMS |
| [La Velle et al.](https://doi.org/10.1080/02607476.2020.1803051) | Europe | United Kingdom | DM | N/S | - | Edu | LMS, SCT, TBT, MPT, AT |
| [Lall & Singh](https://doi.org/10.26452/ijrps.v11iSPL1.2122) | Asia | India | S | 200 | N/S | H&W, Eng, B, ICT | SCT, TBT, MPT |
| [Lassoued, Alhendawi & Bashitialshaaer](https://doi.org/10.3390/educsci10090232) | Africa, Middle East | Iraq, Algeria, Egypt, Palestine | S, T | 400 | N/S | N/S | N/S |
| [Liberman-Martin & Ogba](https://doi.org/10.1021/acs.jchemed.0c00632) | North America | United States | S, T | 55 | UG | NSM | SCT, TBT, AT, LMS |
| [Lima et al.](https://doi.org/10.1152/ADVAN.00131.2020) | S & C America | Brazil | S | 50 | UG | NSM, H&W | KOS, SCT, MPT, AT, SNT |
| [Lin & Gao](https://www.asianjde.org/ojs/index.php/AsianJDE/article/view/448) | Asia | China | S | 1,189 | UG | N/S | SCT, LMS |
| [Lin & Pryor](https://doi.org/10.1007/978-3-030-51968-1_15) | Asia | China | S | 89 | UG | A&H, H&W, Eng, B, SSci, NSM | VW |
| [Littlejohn](https://doi.org/10.46786/ac20.8253) | Europe | United Kingdom | T, SS | 412 | - | N/S | SCT, TBT, MPT |
| [Liu et al.](https://doi.org/10.3390/su12176929) | Asia | China | S, T | 9,382 | UG, PG | N/S | N/S |
| [Longhurst et al.](https://doi.org/10.1002/ase.1967) | Europe | United Kingdom, Ireland | DM | 14 | - | H&W | SCT, VW, LMS |
| [Lundquist, Rice & Widenhorn](https://www.anthologyinc.com/whitepaper/the-pandemic-college-student-experience?download=true#access) | North America | United States | S | 1,143 | UG, PG | N/S | N/S |
| [Lyons et al.](https://doi.org/10.1177/1039856220947945) | Oceania | Australia | S | 297 | PG | H&W | N/S |
| [Lytovchenko & Voronina](https://doi.org/10.18662/RREM/12.2SUP1/291) | Europe | Ukraine | S | 58 | UG | Eng | MOOC |
| [Mahdy](https://doi.org/10.3389/fvets.2020.594261) | Global | Multiple | S | 1,392 | UG, PG | Ag | KOS, SCT, TBT, MPT, SNT, ML, LMS, WCT |
| [Marek et al.](https://doi.org/10.4018/IJDET.20210101.oa3) | Africa, Asia, Europe, Middle East, N. America, S & C America | Multiple | T | 418 | - | A&H, NSM, B, SSci, ICT, Edu, H&W | SCT, TBT, SNT, LMS |
| [Martínez-Garcés & Garcés-Fuenmayor](https://doi.org/10.17081/eduhum.22.39.4114) | S & C America | Colombia | T | 52 | - | N/S | N/S |
| [McRoy et al.](https://doi.org/10.1016/j.acra.2020.08.001) | North America | United States | S, T | 17 | N/S | H&W | SCT, LMS, KOS |
| [Means & Neisler](https://digitalpromise.dspacedirect.org/handle/20.500.12265/98) | North America | United States | S | 1,008 | UG | N/S | SCT, MPT, AT |

Note: UAE = United Arab Emirates, S & C America = South & Central America, T = Teachers/Instructors, S = Students, DM = Department Managers, A = Administrators, SS = Support Staff, UG = Undergraduate, PG = Postgraduate, H&W = Health & Welfare, A&H = Arts & Humanities, NSM = Natural Science, Mathematics & Statistics, Eng = Engineering, Manufacturing & Construction, Edu = Education, B = Business, Administration & Law, N/S = Not specified, SSci = Social Sciences, Journalism & Information, Ag = Agriculture, Forestry, Fisheries & Veterinary, LMS = Learning Management System, SNT = Social networking tools, SCT = Synchronous collaboration tools, TBT = Text-based tools, MPT = Multimedia production tools, AT = Assessment tools, KOT = Knowledge organization & sharing tools, WCT = Website creation tools, ML = Mobile learning, D = Devices used

| **Author** | **Continent** | **Country** | **Participants** | **# Participants** | **Study level** | **Discipline** | **Technology used** |
| --- | --- | --- | --- | --- | --- | --- | --- |
| [Means & Neisler](http://hdl.handle.net/20.500.12265/102) | North America | United States | S | 620 | UG | NSM | SCT, TBT, MPT, AT, D |
| [Milligan](https://doi.org/10.1021/acs.jchemed.0c00686) | North America | United States | S | 35 | UG | NSM | SCT, MPT, AT, LMS |
| [Minghat et al.](https://doi.org/10.18662/rrem/12.1sup2/242) | Asia | Indonesia, Malaysia | S | 136 | N/S | N/S | SCT, TBT, AT, LMS |
| [Mishra, Gupta & Shree](https://doi.org/10.1016/J.IJEDRO.2020.100012) | Asia | India | S, T | 338 | PG | N/S | SCT, TBT, MPT, SNT, LMS |
| [Mitra & Basu](https://doi.org/10.31782/IJCRR.2020.12151) | Asia | India | S | 262 | UG, PG | H&W | SCT, MPT |
| [Mladenova, Kalmukov & Valova](https://doi.org/10.18421/TEM93-42) | Europe | Bulgaria | S, T | 310 | N/S | ICT | SCT, TBT, SNT |
| [Mohammed et al.](https://doi.org/10.1007/s41062-020-00326-7) | Middle East | Oman | S, T | N/S | N/S | N/S | SCT, MPT, SNT, ML, LMS |
| [Molina & Tomás](https://revistas.unlp.edu.ar/econo/article/view/10352) | S & C America | Argentina | S | 96 | PG | N/S | N/S |
| [Mondal & Mondal](https://doi.org/10.25259/IJMS_269_2020) | Asia | India | S | 100 | UG | H&W | SCT, AT, D |
| [Monday et al.](https://doi.org/10.7759/cureus.8558) | North America | United States | S | 89 | UG | H&W | SCT, TBT, AT, LMS |
| [Mondol & Mohiuddin](https://doi.org/10.46291/IJOSPERvol7iss2pp231-247) | Asia | Bangladesh | S, T | 80 | UG | N/S | N/S |
| [Moo](http://scielo.sld.cu/scielo.php?script=sci_arttext&pid=S1990-86442020000500056) | S & C America | Mexico | S | 96 | UG | Edu | N/S |
| [Morales Vaccarezza](http://repositorio.udec.cl/jspui/handle/11594/617) | S & C America | Chile | S, T | 7 | UG | H&W | N/S |
| [Mouchantaf](https://doi.org/10.17507/TPLS.1010.11) | Middle East | Lebanon | T, SS | 50 | - | A&H | SCT, LMS |
| [Mpungose](https://doi.org/10.1057/s41599-020-00603-x) | Africa | South Africa | S | 26 | N/S | Edu | TBT, MPT, LMS |
| [Mukhtar et al.](https://doi.org/10.12669/pjms.36.COVID19-S4.2785) | Asia | Pakistan | S, T | 24 | UG | H&W | SCT, MPT, SNT, LMS |
| [Murphy, Eduljee & Croteau](https://doi.org/10.29333/PR/8485) | North America | United States | S | 148 | UG | Edu, H&W, B, SSci | SCT, LMS |
| [Muthler](https://sites.psu.edu/das202c/files/2020/06/Noah.pdf) | North America | United States | S | 10 | UG | NSM, Eng | N/S |
| [Nassr et al.](https://doi.org/10.1109/ACCESS.2020.3029967) | Asia | Malaysia | S | 284 | UG | ICT | SCT, MPT, SNT |
| [Nenko, Kybalna & Snisarenko](https://doi.org/10.20873/uft.rbec.e8925) | Europe | Ukraine | S | 540 | N/S | N/S | KOS, SCT, TBT, SNT, D |
| [Ng et al.](https://www.researchgate.net/publication/344348102_Business_Teaching_as_Usual_amid_the_COVID-19_Pandemic_A_Case_Study_of_Online_Teaching_Practice_in_Hong_Kong) | Asia | Hong Kong | T | 1 | - | ICT | LMS |
| [Nickerson & Shea](https://doi.org/10.1021/acs.jchemed.0c00674) | North America | United States | S | 63 | UG | NSM | SCT, AT, TBT, MPT |
| [O’Brien et al.](https://doi.org/10.1080/02619768.2020.1823963) | Europe | United Kingdom, Greece, Portugal, Ireland, Finland | T | 12 | - | Edu | N/S |
| [Octaberlina & Muslimin](https://doi.org/10.5430/IJHE.V9N6P1) | Asia | Indonesia | S | 25 | UG | Edu | LMS |
| [Ogrutan & Aciu](https://doi.org/10.18421/TEM92-31) | Europe | Romania | S | 26 | UG | SSci | SCT, AT, LMS |
| [Ohara & Ishimura](https://doi.org/10.37237/110310) | Asia | Japan | S, SS | 5 | UG | A&H | SCT |
| [Olesov et al.](https://doi.org/10.20511/pyr2020.v8nSPE3.709) | Europe | Russia | S | 195 | PG | N/S | LMS |
| [Olmos-Gómez](https://doi.org/10.3390/ijerph17145036) | Europe | Spain | S | 441 | UG | Edu | N/S |
| [Olszewska](http://psjd.icm.edu.pl/psjd/element/bwmeta1.element.psjd-f9ac72d4-40dc-43d6-884f-0c72e497ccb7) | Europe | Poland | S | 734 | UG, PG | N/S | LMS |
| [Omodan](https://dialnet.unirioja.es/descarga/articulo/7495552.pdf%20https:/dialnet.unirioja.es/servlet/extart?codigo=7495552) | Africa | South Africa | S, T, DM, SS | 15 | N/S | N/S | N/S |
| [Pal & Vanijja](https://doi.org/10.1016/j.childyouth.2020.105535) | Asia | India | S | 1,595 | N/S | NSM, Eng, B | SCT |
| [Papouli, Chatzifotiou & Tsairidis](https://doi.org/10.1080/02615479.2020.1807496) | Europe | Greece | S | 550 | UG, PG | SSci | MOOC |
| [Pather et al.](https://doi.org/10.1002/ase.1968) | Oceania | Australia, NZ | T | 18 | - | H&W | SCT, TBT, MPT, AT |
| [Perets et al.](https://doi.org/10.1021/acs.jchemed.0c00879) | North America | United States | S, T, SS | 15 | UG | NSM | SCT, TBT, AT, LMS |
| [Pérez-Jorge et al.](https://doi.org/10.3390/SU12208631) | Europe | Spain | S | 193 | UG, PG | Edu | SCT, TBT |
| [Pérez-López et al.](http://revistas.uned.es/index.php/ried/article/view/27855) | Europe | Spain | S, DM | 548 | UG, PG | Edu, A&H, NSM, H&W, Eng, B, SSci | SCT, MPT, SNT, LMS, WCT, D |
| [Petillion & McNeil](https://doi.org/10.1021/ACS.JCHEMED.0C00733) | North America | Canada | S | 64 | UG | NSM | SCT, MPT, AT |
| [Puljak et al.](https://doi.org/10.1186/s12909-020-02343-7) | Europe | Croatia | S | 2,520 | UG | H&W | SCT, MPT, D |
| [Purwanto et al.](http://www.jcreview.com/fulltext/197-1593970049.pdf?1606218190) | Asia | Indonesia | S | 6 | N/S | Edu, A&H, Eng, B, SSci | N/S |
| [Quezada Castro et al.](http://repositorio.utp.edu.pe/bitstream/UTP/3076/1/Maria%20del%20Pilar%20Quezada%20Castro_Articulo_Revista%20Inclusiones_spa_2020.pdf) | S & C America | Peru | T | 97 | - | N/S | N/S |
| [Radu et al.](https://doi.org/10.3390/ijerph17217770) | Europe | Romania | S | 135 | UG, PG | H&W, Eng | SCT, MPT, AT, SNT, LMS |
| [Rafi, Varghese & Kuttichira](https://doi.org/10.1177/2382120520951795) | Asia | India | S | 364 | UG | H&W | KOS, MPT |
| [Rahali et al.](https://doi.oirg/10.3329/bjms.v19i0.48166) | Africa | Morocco | S | 123 | UG, PG | N/S | SCT, SNT |
| [Rahiem](https://doi.org/10.26803/ijlter.19.6.1) | Asia | Indonesia | S | 80 | UG | SSci | SCT, TBT, MPT, SNT, LMS |
| [Rajab, Gazal & Alkattan](https://doi.org/10.7759/cureus.8966) | Middle East | Saudi Arabia | S, T | 208 | UG, PG | H&W | N/S |
| [Ramachandran & Rodriguez](https://doi.org/10.1021/acs.jchemed.0c00572) | North America | United States | S | 480 | UG | NSM | SCT, TBT, MPT, AT, D, LMS |

Note: UAE = United Arab Emirates, NZ = New Zealand, S & C America = South & Central America, T = Teachers/Instructors, S = Students, DM = Department Managers, A = Administrators, SS = Support Staff, UG = Undergraduate, PG = Postgraduate, H&W = Health & Welfare, A&H = Arts & Humanities, NSM = Natural Science, Mathematics & Statistics, Eng = Engineering, Manufacturing & Construction, Edu = Education, B = Business, Administration & Law, N/S = Not specified, SSci = Social Sciences, Journalism & Information, Ag = Agriculture, Forestry, Fisheries & Veterinary, LMS = Learning Management System, SNT = Social networking tools, SCT = Synchronous collaboration tools, TBT = Text-based tools, MPT = Multimedia production tools, AT = Assessment tools, KOT = Knowledge organization & sharing tools, WCT = Website creation tools, ML = Mobile learning, D = Devices used

| **Author** | **Continent** | **Country** | **Participants** | **# Participants** | **Study level** | **Discipline** | **Technology used** |
| --- | --- | --- | --- | --- | --- | --- | --- |
| [Ranga](https://doi.org/10.1021/acs.jchemed.0c00633) | North America | United States | S | 29 | UG | NSM | SCT, TBT, MPT, LMS |
| [Rapanta et al.](https://doi.org/10.1007/s42438-020-00155-y) | Europe, N. America, Oceania | Spain, Australia, Canada, Switzerland | T | 4 | - | Edu | SCT, TBT, MPT, DAT, AT, LMS |
| [Ravaei et al.](https://doi.org/10.3390/PROCEEDINGS2020054009) | Europe | Spain | S | 84 | UG | H&W | VW |
| [Raza et al.](https://doi.org/10.1177/0735633120960421) | Asia | Pakistan | S | 516 | UG, PG | N/S | LMS |
| [Reyes-Chua et al.](https://doi.org/10.31838/jcr.07.11.41) | Asia | Philippines | T | 15 | - | Edu, A&H, NSM, B, SSci, ICT | SCT, TBT, MPT, LMS |
| [Reynders & Ruder](https://doi.org/10.1021/acs.jchemed.0c00615) | North America | United States | S, T | 127 | UG | NSM | SCT, AT, SNT |
| [Rizun & Strzelecki](https://doi.org/10.3390/ijerph17186468) | Europe | Poland | S | 1,692 | UG, PG | N/S | N/S |
| [Rodríguez Núñez & Leeuwner](https://doi.org/10.1021/j.jchemed.0c00781) | North America | Canada | S | 1,672 | UG | NSM | SCT, TBT, MPT, AT, LMS |
| [Rodríguez-Rodríguez et al.](https://doi.org/10.1021/ACS.JCHEMED.0C00923) | Europe | Spain | S | 306 | UG | NSM | SCT, AT |
| [Roig-Vila et al.](http://revistas.uned.es/index.php/ried/article/view/27519) | Europe | Spain | S | 52 | UG | Edu | SCT, TBT, SNT, LMS, MPT |
| [Roy et al.](https://doi.org/10.7860/JCDR/2020/44869.13797) | Asia | India | S | 182 | UG | H&W | SCT |
| [Rupnow et al.](https://doi.org/10.1021/acs.jchemed.0c00802) | North America | United States | T | 6 | - | NSM | SCT, TBT, MPT, LMS |
| [Sáiz-Manzanares et al.](https://doi.org/10.3390/ijerph17155618) | Europe | Spain | S | 109 | UG | H&W | MPT, DAT, AT, LMS |
| [Sales et al.](https://doi.org/10.3145/epi.2020.jul.23) | Europe | Spain | T | 20 | - | SSci | N/S |
| [Sarju](https://doi.org/10.1021/acs.jchemed.0c00786) | Europe | United Kingdom | S | 44 | UG | NSM | SCT, TBT, MPT, AT, LMS |
| [Sarmiento-Espinoza et al.](https://dialnet.unirioja.es/descarga/articulo/7506222.pdf%20https:/dialnet.unirioja.es/servlet/extart?codigo=7506222) | S & C America | Ecuador | S | 338 | N/S | N/S | LMS |
| [Sarwar et al.](https://doi.org/10.1055/S-0040-1717000) | Asia | Pakistan | S | 1,207 | UG | H&W | TBT, SNT, LMS, D |
| [Savarese et al.](https://doi.org/10.3390/HEALTHCARE8040440) | Europe | Italy | S | 266 | N/S | N/S | N/S |
| [Savcheva & Domuschieva-Rogleva](https://azbuki.bg/wp-content/uploads/2020/09/NSA-Evelina-Savcheva-Galina-Domuschieva.pdf) | Europe | Bulgaria | T | 67 | - | Edu | SCT, TBT, LMS |
| [Schlenz et al.](https://doi.org/10.1186/s12909-020-02266-3) | Europe | Germany | S, T | 277 | N/S | H&W | SCT, TBT, MPT |
| [Schmölz, Geppert & Barberi](https://doi.org/10.21243/mi-02-20-31) | Europe | Austria | S | 412 | N/S | Edu | N/S |
| [Scruggs et al.](https://doi.org/10.1021/acs.jchemed.0c00699) | North America | United States | S | 43 | UG | NSM | SCT, VW |
| [Scull et al.](https://doi.org/10.1080/02607476.2020.1802701) | Oceania | Australia | T | 4 | - | Edu | SCT, TBT, LMS |
| [Sepulveda-Escobar & Morrison](https://doi.org/10.1080/02619768.2020.1820981) | S & C America | Chile | S | 27 | UG | Edu | WCT |
| [Sharadgah & Sa’di](https://doi.org/10.28945/4615) | Middle East | Saudi Arabia | T | 96 | - | N/S | AT, LMS |
| [Shetty et al.](https://doi.org/10.1007/s12070-020-02224-x) | Asia | India | S | 170 | UG | H&W | D |
| [Shim & Lee](https://doi.org/10.1016/j.childyouth.2020.105578) | Asia | South Korea | S | 393 | UG | Edu, A&H, NSM, Eng, B, SSci | D |
| [Shivam & Wagoner](https://arxiv.org/abs/2008.04499) | North America | United States | S | 43 | UG | NSM | SCT, TBT, MPT, VW |
| [Silva et al.](https://doi.org/10.1111/eje.12583) | S & C America | Brazil | S | 142 | UG | H&W | SCT, MPT, D, LMS |
| [Simon et al.](https://doi.org/10.1021/acs.jchemed.0c00778) | North America | United States | S | 41 | UG | A&H, B | TBT, MPT, AT, LMS |
| [Sindiani et al.](https://doi.org/10.1016/J.AMSU.2020.09.036) | Middle East | Jordan | S | 2,212 | UG | H&W | SCT, TBT, MPT |
| [Singal et al.](https://doi.org/10.1007/s00276-020-02615-3) | Asia | India | S | 80 | UG | H&W | D |
| [Singh, Sharma & Paliwal](https://doi.org/10.1108/ITSE-05-2020-0070) | Asia | India | S | 324 | UG, PG | N/S | N/S |
| [Singhi et al.](https://doi.org/10.1007/s13187-020-01863-6) | North America | United States | S | 30 | PG | H&W | SCT, D |
| [Smoyer, O’Brien & Rodriguez-Keyes](https://doi.org/10.1177/0020872820940021) | North America | United States | S | 122 | UG | SSci | SCT |
| [Sobaih, Hasanein & Abu Elnasr](https://doi.org/10.3390/su12166520) | Africa | Egypt | S, T | 613 | UG | N/S | SNT |
| [Sokhulu](https://doi.org/10.1080/14725843.2020.1801384) | Africa | South Africa | S | 5 | PG | Edu, SSci | SCT, TBT, D |
| [Soria et al.](https://conservancy.umn.edu/handle/11299/214934) | North America | United States | S | 7,233 | UG | N/S | N/S |
| [Srivastava et al.](https://doi.org/10.1007/S12262-020-02592-2) | Asia | India | S | 55 | PG | H&W | SCT, TBT |
| [Stukalo & Simakhova](https://doi.org/10.13189/ujer.2020.080846) | Europe | Ukraine | DM | 397 | - | N/S | LMS |
| [Sukendro et al.](https://doi.org/10.1016/J.HELIYON.2020.E05410) | Asia | Indonesia | S | 974 | N/S | H&W | N/S |
| [Sunasee](https://doi.org/10.1021/acs.jchemed.0c00542) | North America | United States | S | 28 | UG | NSM | SCT, LMS |
| [Swartwood](https://sites.psu.edu/files/DanaFormalReport.pdf) | North America | United States | S | 37 | N/S | A&H | SCT, MPT |
| [Syahrin & Salih](https://doi.org/10.24093/awej/vol11no3.3) | Middle East | Oman | S | 32 | UG | A&H | LMS |
| [Syauqi, Munadi & Triyono](https://doi.org/10.11591/IJERE.V9I4.20766) | Asia | Indonesia | S | 56 | N/S | Eng | N/S |
| [Tang et al.](https://doi.org/10.1080/10494820.2020.1817761) | Asia | China | S | 11,088 | UG | Eng | SCT, TBT, MPT |

Note: UAE = United Arab Emirates, NZ = New Zealand, S & C America = South & Central America, T = Teachers/Instructors, S = Students, DM = Department Managers, A = Administrators, SS = Support Staff, UG = Undergraduate, PG = Postgraduate, H&W = Health & Welfare, A&H = Arts & Humanities, NSM = Natural Science, Mathematics & Statistics, Eng = Engineering, Manufacturing & Construction, Edu = Education, B = Business, Administration & Law, N/S = Not specified, SSci = Social Sciences, Journalism & Information, Ag = Agriculture, Forestry, Fisheries & Veterinary, LMS = Learning Management System, SNT = Social networking tools, SCT = Synchronous collaboration tools, TBT = Text-based tools, MPT = Multimedia production tools, AT = Assessment tools, KOT = Knowledge organization & sharing tools, WCT = Website creation tools, ML = Mobile learning, DAT = Data Analytics Tools, D = Devices used

| **Author** | **Continent** | **Country** | **Participants** | **# Participants** | **Study level** | **Discipline** | **Technology used** |
| --- | --- | --- | --- | --- | --- | --- | --- |
| [Tartavulea et al.](https://doi.org/10.24818/EA/2020/55/920) | Europe | Multiple | S, T | 362 | N/S | N/S | KOS, SCT, AT, TBT |
| [Terenko & Ogienko](https://doi.org/10.18662/rrem/12.1sup2/261) | Europe | Ukraine | S, T, SS | 125 | UG | A&H, NSM | SCT, TBT, MPT, AT, LMS |
| [Tigaa & Sonawane](https://doi.org/10.1021/acs.jchemed.0c00554) | Asia, North America | India, United States | S | 150 | UG | NSM | SCT, TBT, MPT, SNT, LMS |
| [Tsekea & Chigwada](https://doi.org/10.1108/DLP-06-2020-0058) | Africa | Zimbabwe | SS | 34 | - | SSci | SCT, MPT, SNT, LMS |
| [Tung Son et al.](https://covidscholar.org/article/5f40df9836aff614e80c12aa) | Asia | Vietnam | S | 2,400 | N/S | B, ICT | AT, LMS, SCT |
| [Utama et al.](https://doi.org/10.30651/JQM.V4I2.5000) | Asia | Indonesia | S | 189 | UG | H&W | SCT, TBT, MPT, LMS |
| [Utomo et al.](https://doi.org/10.18280/isi.250314) | Asia | Indonesia | S | 256 | N/S | A&H, SSci | SCT, TBT, SNT, LMS |
| [Villanueva et al.](https://doi.org/10.1021/acs.jchemed.0c00752) | North America | United States | S, T | 124 | UG | NSM | SCT, MPT, AT |
| [Vlasova et al.](https://doi.org/10.20511/pyr2020.v8nSPE3.719) | Europe | Russia | S, T | 30 T + ? S | UG, PG | Edu | KOS, SCT, TBT, MPT, AT, SNT, VW, WCT |
| [Wang, Zhang & Ye](https://doi.org/10.1002/jdd.12413) | Asia | China | S, T | 8,740 | UG | H&W | SCT, TBT, MPT, MOOC |
| [Wang & East](https://www.clt-international.org/journal/details/info/aMTMu7Mjlk/Constructing-an-Emergency-Chinese-Curriculum-during-the-Pandemic:-A-New-Zealand-Experience) | Oceania | New Zealand | S, T | 163 | UG | A&H | SCT, MPT, AT, LMS, D |
| [Watermeyer et al.](https://doi.org/10.1007/s10734-020-00561-y) | Europe | United Kingdom | T | 1148 | - | Edu, A&H, NSM, H&W, Eng, B, ICT, Ag | N/S |
| [Wiltse et al.](https://www.jehc.eu/index.php/jehc/article/view/143) | North America | United States | S | 230 | N/S | N/S | SCT, TBT, MPT, LMS |
| [Ye et al.](https://doi.org/10.1021/ACS.JCHEMED.0C00724) | Europe | Sweden | S | 43 | UG | NSM | SNT, MPT, AT |
| [Youssef et al.](https://doi.org/10.1021/acs.jchemed.0c00792) | North America | United States | S | 68 | UG | N/S | KOS, SCT, TBT, MPT, AT, VW |
| [Zeeshan, Chaudhry & Khan](https://doi.org/10.36902/sjesr-vol3-iss2-2020(383-396)) | Asia | Pakistan | T | 16 | - | N/S | SCT, TBT, LMS, SNT |
| [Zia](https://doi.org/10.1108/IJILT-05-2020-0089) | Middle East | Saudi Arabia | S | 716 | UG, PG | B | N/S |
| [Ziegler et al.](https://psyarxiv.com/md93x/) | Europe | Germany | S | 1,690 | UG, PG | A&H, H&W, Eng, B, SSci | N/S |
| [Zulkefli, Hashim & Syahrin](https://doi.org/10.30534/IJATCSE/2020/304942020) | Middle East | Oman | S | 22 | N/S | ICT | LMS |

Note: UAE = United Arab Emirates, NZ = New Zealand, S & C America = South & Central America, T = Teachers/Instructors, S = Students, DM = Department Managers, A = Administrators, SS = Support Staff, UG = Undergraduate, PG = Postgraduate, H&W = Health & Welfare, A&H = Arts & Humanities, NSM = Natural Science, Mathematics & Statistics, Eng = Engineering, Manufacturing & Construction, Edu = Education, B = Business, Administration & Law, N/S = Not specified, SSci = Social Sciences, Journalism & Information, Ag = Agriculture, Forestry, Fisheries & Veterinary, LMS = Learning Management System, SNT = Social networking tools, SCT = Synchronous collaboration tools, TBT = Text-based tools, MPT = Multimedia production tools, AT = Assessment tools, KOT = Knowledge organization & sharing tools, WCT = Website creation tools, ML = Mobile learning, DAT = Data Analytics Tools, D = Devices used
