# Supplementary material for: Emergency remote teaching in higher education: mapping the first global online semester
Source: Int J Educ Technol High Educ. 2021 Aug 30;18(1):50. doi: 10.1186/s41239-021-00282-x (PMC8403509; doi:10.1186/s41239-021-00282-x)
Supplement: Supplementary file 2 — Additional file 2: Appendix S2. Educational technology tool typology based on Bower (2016). [file 41239_2021_282_MOESM2_ESM.docx]

# Appendix S2 – Educational technology tool typology based on Bower (2016)

| Text-based tools | Multimodal production tools | Website creation tools | Knowledge organisation and sharing | Data analysis tools |
| --- | --- | --- | --- | --- |
| Discussion forums  Collaborative writing tools  Readings  Newsletter  Text  RSS  Interactive textbook  Annotation tools  Email  Chat  Instant messaging  Wikis | Animations  Tutorials  Recorded lectures  Videos  Podcast/Vodcast  Screencast  Authoring tools  Voice recorder | Blogs  ePortfolios | Cloud storage  Bookmarking  Diary tool in Moodle | Learning analytics dashboard |
| Digital Storytelling tools | Assessment tools | Social networking tools | Synchronous collaboration tools | Mobile learning |
| Storyboards | eAssessment  Quizzes  ARS  Open badges | Social platforms  Microblogging | Audio-Video conferencing | Apps  mLearning |
| Virtual worlds | Learning software | LMS | Devices used | Games |
| Virtual lab  Simulations  Virtual worlds | Language learning software  Presentation software | Learning management systems | Tablets  Hardware  Interactive whiteboards | Games |
