# Supplementary material for: Emergency remote teaching in higher education: mapping the first global online semester
Source: Int J Educ Technol High Educ. 2021 Aug 30;18(1):50. doi: 10.1186/s41239-021-00282-x (PMC8403509; doi:10.1186/s41239-021-00282-x)
Supplement: Supplementary file 3 — Additional file 3: Appendix S3. List of publications (n = 155). [file 41239_2021_282_MOESM3_ESM.docx]

# Appendix C - List of publications (*n* = 155)

| **Journal Name** | ***f*** | **Journal Name** | ***f*** |
| --- | --- | --- | --- |
| Journal of Chemical Education | 36 | Acta Inform Med. | 1 |
| Sustainability | 9 | Advances In Physiology Education | 1 |
| BMC Medial Education | 6 | African Educational Research Journal | 1 |
| European Journal of Teacher Education | 6 | African Identities | 1 |
| Pedagogy | 6 | Amfiteatru Economic | 1 |
| Revista Românească pentru Educaţie Multidimensională | 6 | Arabian Journal of Business and Research Article Management Review | 1 |
| Education Sciences | 4 | Australasian Psychiatry | 1 |
| International Journal of Environmental Research and Public Health | 4 | BMJ Open | 1 |
| Anatomical Sciences Education | 3 | British Journal of Oral and Maxillofacial Surgery | 1 |
| Annals Of Medicine And Surgery | 3 | Chemie Ingenieur Technik | 1 |
| Arab World English Journal | 3 | Creative Education | 1 |
| Asian Journal of Distance Education | 3 | Data in Brief | 1 |
| Children And Youth Services Review | 3 | Digital Library Perspectives | 1 |
| Journal of Education for Teaching | 3 | Digital Promise Global | 1 |
| Journal of Information Technology Education: Research | 3 | Educación y Humanismo | 1 |
| Propósitos y Representaciones | 3 | Education and Information Technologies | 1 |
| Bangladesh Journal of Medical Science | 2 | Educational Assessment, Evaluation and Accountability | 1 |
| Campus Virtuales | 2 | ELT in Focus | 1 |
| Cureus | 2 | European Journal Of Dentistry | 1 |
| Ecology and Evolution | 2 | European Journal of Education Studies | 1 |
| European Journal of Dental Education | 2 | Frontiers In Education | 1 |
| Frontiers in Psychology | 2 | Frontiers in Public Health | 1 |
| Healthcare | 2 | Frontiers in Veterinary Science | 1 |
| IEEE | 2 | Gomal University Journal of Research | 1 |
| Interactive Learning Environments | 2 | Heliyon | 1 |
| Interactive Technology and Smart Education | 2 | Higher Education | 1 |
| International Journal of Academic Research in Business & Social Sciences | 2 | Humanities and Social Sciences Communications | 1 |
| International Journal of Advanced Computer Science and Applications | 2 | Humanities & Social Sciences Reviews | 1 |
| International Journal of Advanced Trends in Computer Science Engineering | 2 | Indian J Otolaryngol Head Neck Surgery | 1 |
| International Journal of Distance Education Technologies | 2 | Indian Journal of Medical Sciences | 1 |
| Journal of Critical Reviews | 2 | Indian Journal Of Surgery | 1 |
| Journal of Interprofessional Care | 2 | Infinity | 1 |
| Pakistan Journal of Medical Sciences | 2 | Information and Learning Sciences | 1 |
| Profesional de la información | 2 | Ingénierie des Systèmes d'Information | 1 |
| Revista Conrado | 2 | Innovative Infrastructure Solutions | 1 |
| RIED. Revista Iberoamericana de Educación a Distancia | 2 | International and Multidisciplinary Journal of Social Sciences | 1 |
| Social Education Research | 2 | International Education & Research Journal | 1 |
| Social Work Education | 2 | International Journal of Advanced Research in Engineering and Technology | 1 |
| Studies in Self-Access Learning Journal | 2 | International Journal of Chinese Language Teaching | 1 |
| TEM Journal | 2 | International Journal of Community Medicine and Public Health | 1 |
| Academic Radiology | 1 | International Journal of Current Research and Review | 1 |
| Access: Contemporary Issues in Education | 1 | International Journal of Educational Research Open | 1 |
| ***Journal Name*** | ***f*** | ***Journal Name*** | ***f*** |
| International Journal Of Evaluation And Research In Education | 1 | Research in Dance Education | 1 |
| International Journal of Higher Education | 1 | Retorika. Jurnal Bahasa, Sastra dan Pengajarannya | 1 |
| International Journal of Language Education | 1 | Revista Brasileira de Educacao do Campo | 1 |
| International Journal of Learning, Teaching and Educational Research | 1 | Revista da Associacao Media Brasileira | 1 |
| International Journal of Research in Pharmaceutical Sciences | 1 | Revista de Educación a Distancia | 1 |
| International Journal of Social, Political and Economic Research | 1 | Revista de la Universidad del Zulia | 1 |
| International Online Journal of Primary Education | 1 | Revista Econo | 1 |
| International Social Work | 1 | Revista Ibérica de Sistemas e Tecnologias de Informação | 1 |
| Journal of Cancer Education | 1 | Revista Inclusiones | 1 |
| Journal of Clinical and Diagnostic Research | 1 | Revista Latinoamericana de Estudios Educativos | 1 |
| Journal of Dental Education | 1 | RISE – International Journal of Sociology of Education | 1 |
| Journal of Education and e-Learning Research | 1 | Saudi Pharmaceutical Journal | 1 |
| Journal of Education and Learning | 1 | Sir Syed Journal of Education & Social Research | 1 |
| Journal of Educational Computing Research | 1 | Sports Biomechanics | 1 |
| Journal of Educational Technology & Online Learning | 1 | Student Experience in the Research University (SERU) Consortium | 1 |
| Journal of Further and Higher Education | 1 | Studies in Language and Education | 1 |
| Journal of Learning for Development | 1 | Surgical and Radiologic Anatomy | 1 |
| Journal of Loss and Trauma | 1 | Surgical Practice | 1 |
| Journal of Medical Education and Curricular Development | 1 | Symmetry | 1 |
| Journal of Pedagogical Sociology and Psychology | 1 | Talent Development & Excellence | 1 |
| Journal of Public Economics | 1 | Teaching Sociology | 1 |
| Journal of Research in Medical and Dental Science | 1 | Technium Social Sciences Journal | 1 |
| Journal of Research on Technology in Education | 1 |  | 1 |
| Journal of Surgical Education | 1 | The International Journal of Information and Learning Technology | 1 |
| Journal of Technology and Teacher Education | 1 | Theory and Practice in Language Studies | 1 |
| Journal of the European Honors Council | 1 | United Kingdom & Ireland Computing Education Research conference | 1 |
| Journal of Voice | 1 | Universal Journal of Educational Research | 1 |
| Jurnal Abiwara | 1 | World Scientific News | 1 |
| Medical Education | 1 |  |  |
| Medical Education Online | 1 |  |  |
| Medienimpulse | 1 |  |  |
| National Institute for Learning Outcomes Assessment | 1 |  |  |
| Nursing Forum | 1 |  |  |
| Online Learning | 1 |  |  |
| Orthodontic Journal of Nepal | 1 |  |  |
| Pedagogical Research | 1 |  |  |
| Physica Medica | 1 |  |  |
| Physics Education | 1 |  |  |
| Plos One | 1 |  |  |
| Polo del Conocimiento | 1 |  |  |
| Postdigital Science and Education | 1 |  |  |
| Proceedings | 1 |  |  |
| Quanun Medika | 1 |  |  |
| Rawal Medical Journal | 1 |  |  |
