# Supplementary material for: Emergency remote teaching in higher education: mapping the first global online semester
Source: Int J Educ Technol High Educ. 2021 Aug 30;18(1):50. doi: 10.1186/s41239-021-00282-x (PMC8403509; doi:10.1186/s41239-021-00282-x)
Supplement: Supplementary file 4 — Additional file 4: Appendix S4. Scope of participant focus. [file 41239_2021_282_MOESM4_ESM.docx]

**Appendix D.** Scope of participant focus

| Participant Focus | *N* Studies | *N* Studies [%] |
| --- | --- | --- |
| Students | 233 | 82.6 |
| Teachers/Instructors | 81 | 28.7 |
| Department Managers | 10 | 3.5 |
| Support Staff | 8 | 2.8 |
| Librarians | 2 | 0.7 |
| IT experts and developers | 1 | 0.4 |
| Policy makers | 1 | 0.4 |

Crosstabulation of participants (*n* = 282)

| **Code** | Students | Teachers/Instructors | Department Managers | Librarians | IT experts and developers | Policy makers | Support Staff |
| --- | --- | --- | --- | --- | --- | --- | --- |
| Students | 233 | 39 | 2 | 0 | 1 | 1 | 5 |
| Teachers/Instructors | 39 | 81 | 5 | 0 | 1 | 1 | 6 |
| Department Managers | 2 | 5 | 10 | 0 | 0 | 0 | 1 |
| Librarians | 0 | 0 | 0 | 2 | 0 | 0 | 0 |
| IT experts and developers | 1 | 1 | 0 | 0 | 1 | 1 | 0 |
| Policy makers | 1 | 1 | 0 | 0 | 1 | 1 | 0 |
| Support Staff | 5 | 6 | 1 | 0 | 0 | 0 | 8 |
