# Supplementary material for: Emergency remote teaching in higher education: mapping the first global online semester
Source: Int J Educ Technol High Educ. 2021 Aug 30;18(1):50. doi: 10.1186/s41239-021-00282-x (PMC8403509; doi:10.1186/s41239-021-00282-x)
Supplement: Supplementary file 5 — Additional file 5: Appendix S5. Number of participants per study (n = 282). [file 41239_2021_282_MOESM5_ESM.docx]

**Appendix E.** Number of participants per study (*n* = 282)

| Number of participants | *N* studies | *N* studies [%] |
| --- | --- | --- |
| 1-25 | 46 | 16.3 |
| 26-50 | 28 | 9.9 |
| 51-100 | 49 | 17.4 |
| 101-200 | 44 | 15.6 |
| 201-300 | 19 | 6.7 |
| 301-400 | 21 | 7.4 |
| More | 71 | 25.2 |
| Not clear | 4 | 1.4 |
