# Supplementary material for: Emergency remote teaching in higher education: mapping the first global online semester
Source: Int J Educ Technol High Educ. 2021 Aug 30;18(1):50. doi: 10.1186/s41239-021-00282-x (PMC8403509; doi:10.1186/s41239-021-00282-x)
Supplement: Supplementary file 6 — Additional file 6: Appendix S6. Crosstabulation of disciplines (n = 282). [file 41239_2021_282_MOESM6_ESM.docx]

**Appendix F.** Crosstabulation of disciplines (*n* = 282)

|  | **A&H** | **BAL** | **EDU** | **EMC** | **H&W** | **ICT** | **NSMS** | **SSJI** | **Unclear** | **Other** | **AFFV** |
| --- | --- | --- | --- | --- | --- | --- | --- | --- | --- | --- | --- |
| **A&H** | 40 | 17 | 12 | 12 | 11 | 7 | 17 | 14 | 0 | 1 | 1 |
| **BAL** | 17 | 26 | 12 | 15 | 11 | 10 | 13 | 13 | 0 | 2 | 1 |
| **EDU** | 12 | 12 | 45 | 9 | 8 | 6 | 11 | 12 | 0 | 1 | 1 |
| **EMC** | 12 | 15 | 9 | 30 | 12 | 6 | 14 | 10 | 0 | 2 | 1 |
| **H&W** | 11 | 11 | 8 | 12 | 77 | 3 | 12 | 9 | 0 | 1 | 1 |
| **ICT** | 7 | 10 | 6 | 6 | 3 | 16 | 5 | 5 | 0 | 1 | 1 |
| **NSMS** | 17 | 13 | 11 | 14 | 12 | 5 | 68 | 11 | 0 | 1 | 1 |
| **SSJI** | 14 | 13 | 12 | 10 | 9 | 5 | 11 | 26 | 0 | 2 | 1 |
| **Unclear** | 0 | 0 | 0 | 0 | 0 | 0 | 0 | 0 | 66 | 0 | 0 |
| **Other** | 1 | 2 | 1 | 2 | 1 | 1 | 1 | 2 | 0 | 2 | 0 |
| **AFFV** | 1 | 1 | 1 | 1 | 1 | 1 | 1 | 1 | 0 | 0 | 2 |

*Note.* A&H = Arts & Humanities, BAL = Business, Administration & Law, EDU = Education, EMC = Engineering, Manufacturing & Construction, H&W = Health & Welfare, NSMS = Natural Sciences, Mathematics & Statistics, SSJI = Social Sciences, Journalism & Information, AFFV = Agriculture, Forestry, Fisheries & Veterinary
