# Supplementary material for: Emergency remote teaching in higher education: mapping the first global online semester
Source: Int J Educ Technol High Educ. 2021 Aug 30;18(1):50. doi: 10.1186/s41239-021-00282-x (PMC8403509; doi:10.1186/s41239-021-00282-x)
Supplement: Supplementary file 7 — Additional file 7: Appendix S7. Data collection methods (n = 282). [file 41239_2021_282_MOESM7_ESM.docx]

**Appendix G.** Data collection methods (*n* = 282)

| Data Collection | *N* Studies | *N* Studies [%] |
| --- | --- | --- |
| (Online) survey | 234 | 83.0 |
| Semi-structured interviews | 41 | 14.5 |
| Student grades | 21 | 7.4 |
| Analytics data | 16 | 5.7 |
| Focus group | 10 | 3.5 |
| Interviews (not specified) | 10 | 3.5 |
| Documents/text | 9 | 3.2 |
| In-depth interviews | 3 | 1.1 |
| Field notes | 2 | 0.7 |
| Discussion board | 1 | 0.4 |
| Observation | 1 | 0.4 |
| Unclear | 1 | 0.4 |
| Expert interviews | 1 | 0. 4 |

Crosstabulation of data collection methods

|  | **Survey** | **FG** | **SSI** | **D/T** | **Grades** | **AD** | **DB** | **I (U)** | **FN** | **IDI** | **OBS** | **EI** |
| --- | --- | --- | --- | --- | --- | --- | --- | --- | --- | --- | --- | --- |
| **Survey** | 234 | 6 | 16 | 4 | 14 | 8 | 1 | 6 | 1 | 2 | 0 | 0 |
| **FG** | 6 | 10 | 3 | 1 | 0 | 0 | 0 | 0 | 0 | 0 | 0 | 0 |
| **SSI** | 16 | 3 | 41 | 2 | 2 | 1 | 0 | 0 | 0 | 0 | 0 | 0 |
| **D/T** | 4 | 1 | 2 | 9 | 0 | 1 | 0 | 2 | 0 | 0 | 0 | 0 |
| **Grades** | 14 | 0 | 2 | 0 | 21 | 4 | 0 | 0 | 0 | 0 | 0 | 0 |
| **AD** | 8 | 0 | 1 | 1 | 4 | 16 | 0 | 1 | 1 | 0 | 0 | 0 |
| **DB** | 1 | 0 | 0 | 0 | 0 | 0 | 1 | 0 | 0 | 0 | 0 | 0 |
| **I (U)** | 6 | 0 | 0 | 2 | 0 | 1 | 0 | 10 | 1 | 0 | 0 | 0 |
| **FN** | 1 | 0 | 0 | 0 | 0 | 1 | 0 | 1 | 2 | 0 | 0 | 0 |
| **IDI** | 2 | 0 | 0 | 0 | 0 | 0 | 0 | 0 | 0 | 3 | 0 | 0 |
| **OBS** | 0 | 0 | 0 | 0 | 0 | 0 | 0 | 0 | 0 | 0 | 1 | 0 |
| **EI** | 0 | 0 | 0 | 0 | 0 | 0 | 0 | 0 | 0 | 0 | 0 | 1 |

*Note.* SSI = Semi-structured interviews, AD = Analytics data, FG = Focus group, I (U) = Interviews (not specified), D/T = Documents/Text, IDI = In-depth interviews, FN = Field notes, DB = Discussion board, OBS = Observation, EI = Expert interviews
