# Supplementary material for: Emergency remote teaching in higher education: mapping the first global online semester
Source: Int J Educ Technol High Educ. 2021 Aug 30;18(1):50. doi: 10.1186/s41239-021-00282-x (PMC8403509; doi:10.1186/s41239-021-00282-x)
Supplement: Supplementary file 8 — Additional file 8: Appendix S8. Data analysis methods (n = 282) [file 41239_2021_282_MOESM8_ESM.docx]

**Appendix H.** Data analysis methods (*n* = 282)

| Data analysis method | *N* Studies | *N* Studies [%] |
| --- | --- | --- |
| Descriptive statistics | 227 | 80.5 |
| Qualitative analysis | 86 | 30.5 |
| Inferential statistics | 81 | 28.7 |
| Correlation analysis | 32 | 11.3 |
| Not explicitly mentioned in the article | 23 | 8.2 |
| Psychometric analysis | 3 | 1.1 |
| Cluster analysis | 2 | 0.7 |
| Machine learning algorithms | 1 | 0.4 |
| Analytic Hierarchy Process (AHP); Technique for Order Preference by Similarity to Ideal Solution (TOPSIS) | 1 | 0.4 |

Crosstabulation of data analysis methods

|  | **QA** | **DS** | **IS** | **CA** | **CLU** | **N/M** | **PA** | **MLA** | **AHP** |
| --- | --- | --- | --- | --- | --- | --- | --- | --- | --- |
| **QA** | 86 | 47 | 15 | 2 | 2 | 6 | 1 | 0 | 0 |
| **DS** | 47 | 227 | 72 | 26 | 2 | 15 | 2 | 0 | 1 |
| **IS** | 15 | 72 | 81 | 13 | 2 | 1 | 3 | 1 | 0 |
| **CA** | 2 | 26 | 13 | 32 | 0 | 0 | 0 | 0 | 0 |
| **CLU** | 2 | 2 | 2 | 0 | 2 | 0 | 0 | 0 | 0 |
| **N/M** | 6 | 15 | 1 | 0 | 0 | 23 | 0 | 0 | 0 |
| **PA** | 1 | 2 | 3 | 0 | 0 | 0 | 3 | 0 | 0 |
| **MLA** | 0 | 0 | 1 | 0 | 0 | 0 | 0 | 1 | 0 |
| **AHP** | 0 | 1 | 0 | 0 | 0 | 0 | 0 | 0 | 1 |

*Note.* QA = Qualitative analysis, DS = Descriptive statistics, IS = Inferential statistics, CA = Correlational analysis, CLU = Cluster analysis, N/M = Not explicitly described in the study, PA = Psychometric analysis, MLA = Machine learning algorithms, AHP = Analytic Hierarchy Process
