# Supplementary material for: Emergency remote teaching in higher education: mapping the first global online semester
Source: Int J Educ Technol High Educ. 2021 Aug 30;18(1):50. doi: 10.1186/s41239-021-00282-x (PMC8403509; doi:10.1186/s41239-021-00282-x)
Supplement: Supplementary file 9 — Additional file 9: Appendix S9. Terminology used to describe teaching and learning during the pandemic (n = 282) [file 41239_2021_282_MOESM9_ESM.docx]

**Appendix I.** Terminology used to describe teaching and learning during the pandemic (*n* = 282)

| Terms used | *N* Studies | *N* Studies [%] |
| --- | --- | --- |
| Online learning | 58 | 20.6% |
| e-Learning | 52 | 18.4% |
| Distance learning | 50 | 17.7% |
| Online teaching | 33 | 11.7% |
| Online education | 18 | 6.4% |
| not specified | 17 | 6.0% |
| Internet Web-Based Learning | 16 | 5.7% |
| Emergency remote teaching | 15 | 5.3% |
| Remote learning | 15 | 5.3% |
| Computer-Based Learning | 8 | 2.8% |
| Distance education | 8 | 2.8% |
| Digital learning | 7 | 2.5% |
| Online classes | 7 | 2.5% |
| Self-instruction | 7 | 2.5% |
| Multimedia-Based Learning | 6 | 2.1% |
| Blended learning | 5 | 1.8% |
| Educación virtual | 5 | 1.8% |
| Online instruction | 4 | 1.4% |
| Online assessment | 3 | 1.1% |
| Remote instruction | 3 | 1.1% |
| Virtual Classes | 3 | 1.1% |
| Enseñanza virtual | 3 | 1.1% |
| Digital teaching | 2 | 0.7% |
| Distance teaching | 2 | 0.7% |
| Emergency remote online learning | 2 | 0.7% |
| Online classroom | 2 | 0.7% |
| Virtual learning | 2 | 0.7% |
| Enseñanza remota de emergencia | 2 | 0.7% |
| Enseñanza a distancia | 2 | 0.7% |
| Docencia virtual | 2 | 0.7% |
| Remote teaching | 2 | 0.7% |
| Cloud-based | 1 | 0.4% |
| Computer-supported collaborative learning | 1 | 0.4% |
| digital education | 1 | 0.4% |
| digital higher education | 1 | 0.4% |
| Digital pedagogy | 1 | 0.4% |
| Emergency distance education (EDE) | 1 | 0.4% |
| Emergency distance learning | 1 | 0.4% |
| Emergency online education | 1 | 0.4% |
| Emergency remote education | 1 | 0.4% |
| Emergency remote support | 1 | 0.4% |
| Emerging Technology | 1 | 0.4% |
| e-assessment | 1 | 0.4% |
| e-Teaching | 1 | 0.4% |
| E-Technology | 1 | 0.4% |
| Home learning | 1 | 0.4% |
| information and communications and media technology (ICMT) | 1 | 0.4% |
| MOOCs | 1 | 0.4% |
| Terms used | N Studies | N Studies [%] |
| Net based learning | 1 | 0.4% |
| online course | 1 | 0.4% |
| Online delivery | 1 | 0.4% |
| Online examination | 1 | 0.4% |
| Online synchronous teaching | 1 | 0.4% |
| Remote assessment | 1 | 0.4% |
| Remote classes | 1 | 0.4% |
| Remote E-exams | 1 | 0.4% |
| Remote labs | 1 | 0.4% |
| Virtual education | 1 | 0.4% |
| Virtual teaching | 1 | 0.4% |
| Enseñanza online | 1 | 0.4% |
| Educación a distancia | 1 | 0.4% |
| Docencia remota de emergencia | 1 | 0.4% |
| Formación a distancia | 1 | 0.4% |
| Educación online | 1 | 0.4% |
| VirtualizaciÃ³n de cursos | 1 | 0.4% |
| Enseñanza semipresencial | 1 | 0.4% |
| Enseñanza digital | 1 | 0.4% |
| Educación en línea | 1 | 0.4% |
| Virtual classrooms | 1 | 0.4% |
| Self-access learning | 1 | 0.4% |
| Online-blended learning | 1 | 0.4% |
