# Supplementary material for: Emergency remote teaching in higher education: mapping the first global online semester
Source: Int J Educ Technol High Educ. 2021 Aug 30;18(1):50. doi: 10.1186/s41239-021-00282-x (PMC8403509; doi:10.1186/s41239-021-00282-x)
Supplement: Supplementary file 10 — Additional file 10: Appendix S10. Technology used based on Bower’s (2016) typology (n = 282) [file 41239_2021_282_MOESM10_ESM.docx]

**Appendix J.** Technology used based on Bower’s (2016) typology (*n* = 282)

| Data analysis method | *N* Studies | *N* Studies [%] |
| --- | --- | --- |
| Synchronous collaboration tools | 146 | 51.8 |
| LMS | 117 | 41.8 |
| Multimodal production tools | 98 | 34.8 |
| Text-based tools | 90 | 31.9 |
| Assessment tools | 63 | 22.3 |
| Not specified | 58 | 20.6 |
| Social networking tools | 40 | 14.2 |
| Devices used | 40 | 14.2 |
| Knowledge organisation & sharing tools | 18 | 6.4 |
| Virtual worlds | 15 | 5.3 |
| MOOCs | 6 | 2.1 |
| Website creation tools | 5 | 1.8 |
| Data analytics tools | 2 | 0.7 |
| Mobile learning | 2 | 0.7 |
| Games | 1 | 0.4 |
